# Sulfur-oxidizing symbionts colonize the digestive tract of their lucinid hosts

**DOI:** 10.1093/ismejo/wrae200

**Published:** 2024-10-10

**Authors:** Cristina M Alcaraz, Joana Séneca, Martin Kunert, Christopher Pree, Marta Sudo, Jillian M Petersen

**Affiliations:** University of Vienna, Centre for Microbiology and Environmental Systems Science, Division of Microbial Ecology, Djerassiplatz 1, 1030 Vienna, Austria; Doctoral School in Microbiology and Environmental Science, University of Vienna, Djerassiplatz 1, 1030 Vienna, Austria; University of Vienna, Centre for Microbiology and Environmental Systems Science, Division of Microbial Ecology, Djerassiplatz 1, 1030 Vienna, Austria; Joint Microbiome Facility of the Medical University of Vienna and the University of Vienna, Djerassiplatz 1, 1030 Vienna, Austria; University of Vienna, Centre for Microbiology and Environmental Systems Science, Division of Microbial Ecology, Djerassiplatz 1, 1030 Vienna, Austria; University of Vienna, Centre for Microbiology and Environmental Systems Science, Division of Microbial Ecology, Djerassiplatz 1, 1030 Vienna, Austria; University of Vienna, Centre for Microbiology and Environmental Systems Science, Division of Microbial Ecology, Djerassiplatz 1, 1030 Vienna, Austria; Center for Electromicrobiology, Section for Microbiology, Department of Biology, Aarhus University, Ny Munkegade 114, 8000 Aarhus C, Denmark; University of Vienna, Centre for Microbiology and Environmental Systems Science, Division of Microbial Ecology, Djerassiplatz 1, 1030 Vienna, Austria; Doctoral School in Microbiology and Environmental Science, University of Vienna, Djerassiplatz 1, 1030 Vienna, Austria

**Keywords:** holobiont, lucinids, marine chemosymbiosis, animal-microbe symbiosis

## Abstract

Like many marine invertebrates, marine lucinid clams have an intimate relationship with beneficial sulfur-oxidizing bacteria located within specialized gill cells known as bacteriocytes. Most previous research has focused on the symbionts in the gills of these (and other) symbiotic bivalves, often assuming that the symbionts only persistently colonize the gills, at least in the adult stage. We used 16S rRNA gene sequencing and digital polymerase chain reaction with symbiont-specific primers targeting the *soxB* gene on the foot, mantle, visceral mass, and gills of the lucinid clam *Loripes orbiculatus*. We also used fluorescence in situ hybridization with symbiont-specific probes to examine symbiont distribution at the level of the whole holobiont. Despite 40 years of research on these symbioses, we detected previously unknown populations of symbiont cells in several organs, including the digestive tract. As in the well-studied gills, symbionts in the digestive tract may be housed within host cells. A 14-month starvation experiment without hydrogen sulfide to power symbiont metabolism caused a larger reduction in symbiont numbers in the gills compared to the visceral mass, raising the possibility that symbionts in the digestive tract are persistent and may have a distinct physiology and role in the symbiosis compared with the gill symbionts. Our results highlight the unexpectedly complex relationships between marine lucinid clams and their symbionts and challenge the view that chemosynthetic symbionts are restricted to the gills of these hosts.

## Introduction

Symbiotic partnerships between hosts and microbes play a crucial role in shaping the physiology, ecology, and evolutionary trajectories of life on Earth [1]. All known members of the marine bivalve family Lucinidae host an ancient symbiosis with beneficial sulfur-oxidizing bacteria from the family Sedimenticolaceae, with one exception [2, 3]. The symbionts are acquired horizontally from the surrounding environment and housed within specialized cells called bacteriocytes in the gill epithelia [1]. This is a nutritional symbiosis; symbionts use the energy they gain from sulfide oxidation to fix carbon (C), providing for their own and up to 80% of their host’s nutritional needs [4]. The symbionts are also capable of nitrogen (N) fixation, which may provide a source of N for the symbiosis [5, 6]. These chemosynthetic symbioses allow lucinids to flourish in sulfidic sediments such as those in and around seagrass meadows [7]. Furthermore, by oxidizing hydrogen sulfide to non-toxic sulfur species, chemosynthetic symbioses can support the health of key coastal “blue carbon” ecosystems [8].

Some animals that host chemosynthetic symbionts, such as pogonophoran and oligochete worms, have completely lost their digestive tract, at least in the (symbiotic) adult stage, and presumably rely on the symbionts for their entire nutritional needs [9]. Most bivalves, in contrast, including the lucinids and vesicomyid clams, have maintained a digestive tract with reduced complexity and functionality compared to their non-symbiotic relatives, even though some of these symbioses have a much longer evolutionary history than the gutless worms [10, 11]. The study of microbiomes associated with animal (including human) digestive tracts is a major research field, but in chemosynthetic symbioses, research has been mainly limited to the gills, with some exceptions [4, 11–14]. It is currently unknown whether these animals also host a gut microbiome like their relatives that rely on filter- or deposit-feeding for nutrition [15] or if their intimate gill symbioses influence their interactions with other microorganisms, including those in the gut. We aimed to address this knowledge gap by investigating host-microbe associations in the marine lucinid clam *Loripes orbiculatus* at the level of the whole holobiont through microscopy, amplicon sequencing, digital polymerase chain reaction (dPCR), and experimental manipulation of symbiont load.

## Results and discussion

**Figure 1:**
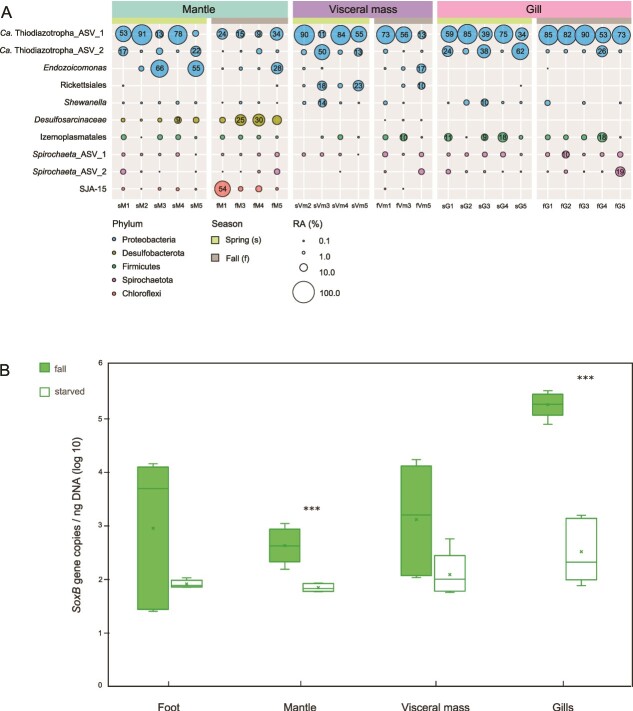
(A) *Ca.* thiodiazotropha ASVs comprise the majority of the reads recovered by 16S rRNA gene sequencing (V3-V4 region) in all organs of fresh *Loripes orbiculatus*. Bubble plots show relative abundance (RA%) of the top 10 most abundant ASVs detected in all samples collected during two seasons: Spring and Fall. Only samples with at least 350 reads are shown; samples below this threshold have been removed. (B) Symbiont-encoded *soxB* gene measured in fresh and starved *L. orbiculatus* tissues*.* Box plots of dPCR analysis show the log 10 values of gene copies/ng DNA in the foot, mantle, visceral mass, and gills. The sample size for each tissue is *n* = 5; per treatment. Statistical significance is displayed at: *P >* .05 = ^*^, *P >* .01 = ^**^, *P* > .001 = ^***^.

To investigate the microbiome associated with different lucinid organs in *Loripes orbiculatus*, we sequenced the 16S rRNA gene from separated gill, mantle, visceral mass (includes digestive tract), and foot organs of 10 individuals sampled across two different seasons (Fig. 1; Fig. S1). As seen in previous studies, the diversity of the gill microbiome was limited [16, 17] and was dominated by two amplicon sequence variants (ASVs) (comprising between 77% and 96% of the reads/sample) assigned to the intracellular symbiont genus *Ca.* thiodiazotropha. These ASVs were identical to those of previously described symbionts found in the gills of lucinid clams at this site [5]. Additionally, these two *Ca.* thiodiazotropha ASVs also made up the majority of reads in all mantle (16%–91%) and visceral mass (14%–95%) samples (Fig. 1), although neither of these organs were previously known to host chemosynthetic symbionts. The same ASVs were also detectable in foot samples, but because the total number of reads recovered from this organ was so low (below 350 reads), they were excluded from further analyses. Other microorganisms detected in different organs included *Endozoicomonas*, *Rickettsiales*, *Shewenella*, *Izemoplasmatales*, and Spirochetes. *Shewenella* and *Izemoplasmatales* are common in seawater, sediments, and other invertebrate animals [18, 19]. *Endozoicomonas* and Spirochetes have been identified in the gills of several lucinid species [16, 17], and ultrastructural studies have shown intracellular structures described as *Rickettsia*-like or *Chlamydia*-like bacteria in the digestive organs of *Loripes orbiculatus* [4, 12].

**Figure 2:**
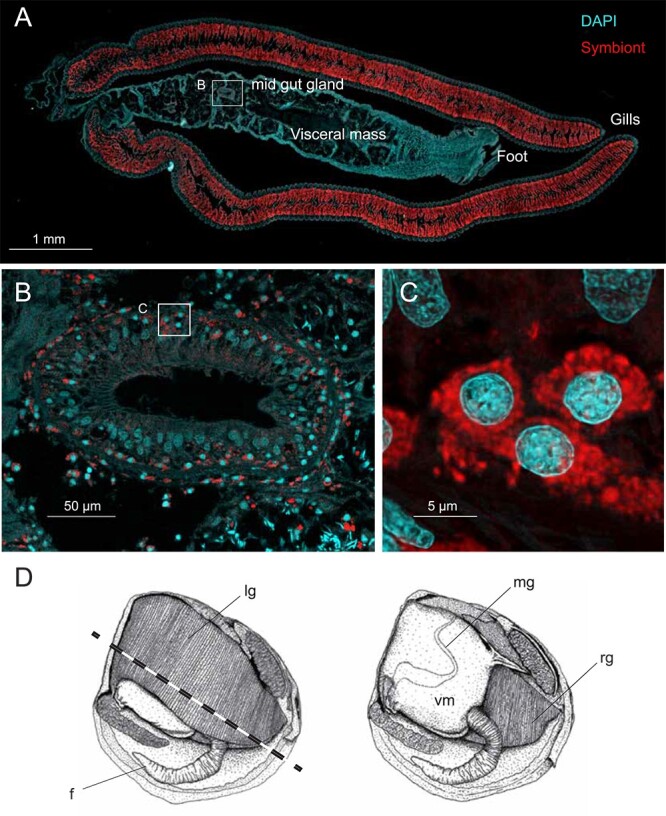
*Ca.* thiodiazotropha symbionts colonize the gills and digestive tract of the bivalve *Loripes orbiculatus*. (A) Whole body section of *L. orbiculatus* with the following organs labeled: visceral mass, foot, and gills. The outline (labeled B) highlights the mid-gut gland in the visceral mass. Red: symbiont probe, cyan: DAPI. (B) Mid-gut gland with symbionts surrounding the perimeter centralized around host nuclei. The outline (labeled C) highlights three individual host bacteriocytes with symbionts. (C) Three host bacteriocytes, with symbionts surrounding host nuclei. (D) Illustration of the lucinid anatomy based on the species *Rasta thiophila*, modified from (11). Abbreviations: f, foot; lg, left gill; vm, visceral mass; mg, mid-gut; rg, right gill. The dashed line in the left illustration indicates the orientation of sectioning through the host body. In the left illustration the right gill (rg) is removed for a better depiction of visceral mass (vm) with mid-gut (mg).

Because the gill symbionts are so numerous, it is possible that the *Ca.* thiodiazotropha reads detected in other organs were due to contamination, even though we were as careful as possible during dissection and meticulously washed each sample separately before freezing the samples for DNA extraction. We therefore used fluorescence *in situ* hybridization (FISH) with probes (Table S1) specific to the gill symbionts (Table S2) on whole body sections. As anticipated, gill bacteriocytes of the lucinids were densely populated with symbiotic bacteria (Fig. 2; *n* = 10). We also detected FISH signals in and around the digestive tract within the visceral mass (Fig. 2; Fig. S3), which provided visual support for the presence of symbiont cells in organs other than the gills. These signals were not seen in FISH experiments with a negative control probe (Fig. S3). In dual FISH analyses with a symbiont-specific and a general bacterial probe, most signals from the general probe overlapped with the specific probe, which is consistent with the sequencing results where most reads were from *Ca.* thiodiazotropha (Fig. S3). We did not detect any symbiont cells with FISH in the mantle or in the foot; therefore, the detected reads could have been a product of contamination or possibly symbiont DNA in circulating outer membrane vesicles, as is known from other symbioses [20]. Bacterial cells, if present, may be so sparsely distributed in these organs that the chance of finding them through thin sectioning was too low. Given that FISH signals from the symbiont-specific probe mostly overlapped with those from the general bacterial probe, it seems likely that these bivalves, unlike those that rely entirely on filter feeding, do not host a highly diverse gut microbiome, which is supported by our 16S rRNA gene sequencing results that revealed a limited diversity of microorganisms in the visceral mass (Fig. 1). Depriving lucinid clams of the hydrogen sulfide that powers symbiont chemosynthesis results in loss of the gill symbiont population [21]. We kept *L. orbiculatus* in well-washed sediment deprived of sulfide for 12 to 14 months. dPCR (Fig. 1) and FISH (Figs S2 and S3) confirmed that these individuals had far fewer gill symbionts than those freshly collected. In contrast, the prominent symbiont population in the visceral mass appeared similar in FISH images of starved and fresh individuals (Figs S2 and S4; *n* = 10 per treatment), and quantitative analyses (Fig. 1B; *n* = 5; per treatment) could not detect any significant difference between symbiont-specific *soxB* copy numbers in the visceral mass of fresh vs. starved individuals. This shows that the digestive tract symbiont population may be maintained even without sulfide provision. Additionally, symbiont-bearing host cells in the digestive tract appear larger and more widely dispersed throughout the tissues (Figs S4 and S5) compared to the densely populated bacteriocytes in the gills (Fig. S3). This distinct cell size and distribution difference suggests that symbionts may play varying roles in different tissues.

Although whole genome sequences would be necessary to show that the two symbiont genotypes we detected in multiple organs are the same symbionts, we could show that identical *Ca.* thiodiazotropha ASVs and symbiont-specific *soxB* genes can be detected outside the gills. The exclusive focus of so much previous research on symbionts in the gills is understandable; symbiont distribution and proliferation must be tightly controlled to avoid host overgrowth and the breakdown of the symbiosis. In a weevil that hosts intracellular, nutritional symbionts in a discrete organ, the bacteriome, symbionts are only found in other parts of the host’s body when the host antimicrobial peptide coleoptericin-A is experimentally silenced [22]. Without this control mechanism, symbiont cell division is left unchecked, resulting in infiltration of typically non-symbiont-containing host tissues. Could the presence of sulfur-oxidizing symbionts in the *L. orbiculatus* digestive tract be due to a lack of host control, allowing the symbionts to colonize outside the gills? There are a few possible alternative explanations. Firstly, bacteriocytes from the gill could be sloughed off, transported to the mouth, and ingested. Transport from gill epithelial cells to the gut has been proposed for gill symbiont-encoded cellulases in wood-boring bivalves [23]. In lucinids, it is possible that after ingestion, the bacterial symbionts may survive digestion and be taken up by phagocytic cells such as hemocytes, which can migrate across epithelia. Intracellular sponge symbionts encode eukaryotic-like proteins (ELPs) that enable them to survive phagocytosis [24]. Although ELPs have not been identified in the genomes of lucinid symbionts, they could employ a similar mechanism to survive within cells in the gut, mediated by different proteins. Alternatively, the symbionts in the gut may be a naturally occurring resident population with a distinct role and metabolism compared with the gill symbionts. This contrasts with the intracellular symbionts of *Bathymodiolus* mussels, which colonize all tissues but only in the juvenile stage [14]. The available lucinid symbiont genomes revealed a complete tricarboxylic acid cycle as well as tripartite ATP-independent periplasmic transporters for the uptake of organic compounds, highlighting their metabolic flexibility beyond chemolithoautotrophy, particularly their potential to grow heterotrophically from compounds possibly provided by the host [5]. We currently have no gene expression or metabolic activity data for the symbionts in the digestive tract. However, the results from the starvation experiment are consistent with the theory that the symbionts in the gut are part of a resident population with a distinct function, as they appeared to remain stable and detectable after more than 1 year of sulfide starvation, whereas the gill symbiont population declined massively (Fig. 1). The gut population may be a source of symbionts to the gill, possibly explaining how the gills of experimentally starved animals become fully repopulated a couple of days after replacement into the natural environment [25]. Indeed, symbiont cell division is thought to be inhibited in the gill [26] and must therefore come from the environment or a rapidly dividing source population elsewhere in the host’s body. While studies have shown that lucinids can reacquire symbionts after prolonged periods of sulfide starvation, the source of symbionts remains unclear. They could come from the environment, such as surrounding sediments, or they may be retained and reacquired from the host’s tissues. These two potential modes of symbiont acquisition, environmental and host-based, may even co-occur, providing lucinids with greater flexibility in maintaining their symbiotic relationships.

## Conclusions

Previous studies investigating chemosynthetic symbiosis in marine invertebrates have focused mainly on the first detected symbiont-housing organ, the gills. Here, we show that there is a persistent population of sulfur-oxidizing symbionts outside the gills in the digestive tract of adult hosts. Our study raises several questions about host–microbe interactions in lucinid clams and possibly other chemosynthetic symbioses if such associations across the whole body are widespread. Studies across diverse animal taxa reveal that symbionts may have varied roles throughout different stages of host development, shaping not only physiological but also immunological processes [27]. Because the digestive tract develops before the gills, it is intriguing to speculate that it could be the initial site of colonization and a source of symbionts to the gill, where they play a major role in nutrient provision throughout the host’s lifetime. In general, the emerging field of developmental symbiosis is shedding new light on the processes underlying the establishment and evolution of host-microbe relationships. Our study highlights the importance of investigating these interactions at the level of the entire holobiont.

## Supplementary Material

FigS1_BP2_wrae200

Fig_S2_gill_gut_stvd_wrae200

FISH_revFigS3_wrae200

FigS4AlphaBetaRev2_wrae200

FigS5_fresh_gut_wrae200

Sup_SOX_col_no_images_wrae200

TableS3_ProbeTest_results_wrae200

FISH_revFigS4_wrae200

## Data Availability

The 16S rRNA gene amplicon sequencing datasets generated during the current study are available in the NCBI BioProject repository (https://www.ncbi.nlm.nih.gov/bioproject/), with links to BioProject accession number PRJNA1085550. Supplementary TestProbe data are included in the supplementary information files for this published article.
