# Supplementary material for: Sulfur-oxidizing symbionts colonize the digestive tract of their lucinid hosts
Source: ISME J. 2024 Oct 10;18(1):wrae200. doi: 10.1093/ismejo/wrae200 (PMC11549920; doi:10.1093/ismejo/wrae200)
Supplement: FigS1_BP2_wrae200 [file figs1_bp2_wrae200.pdf]

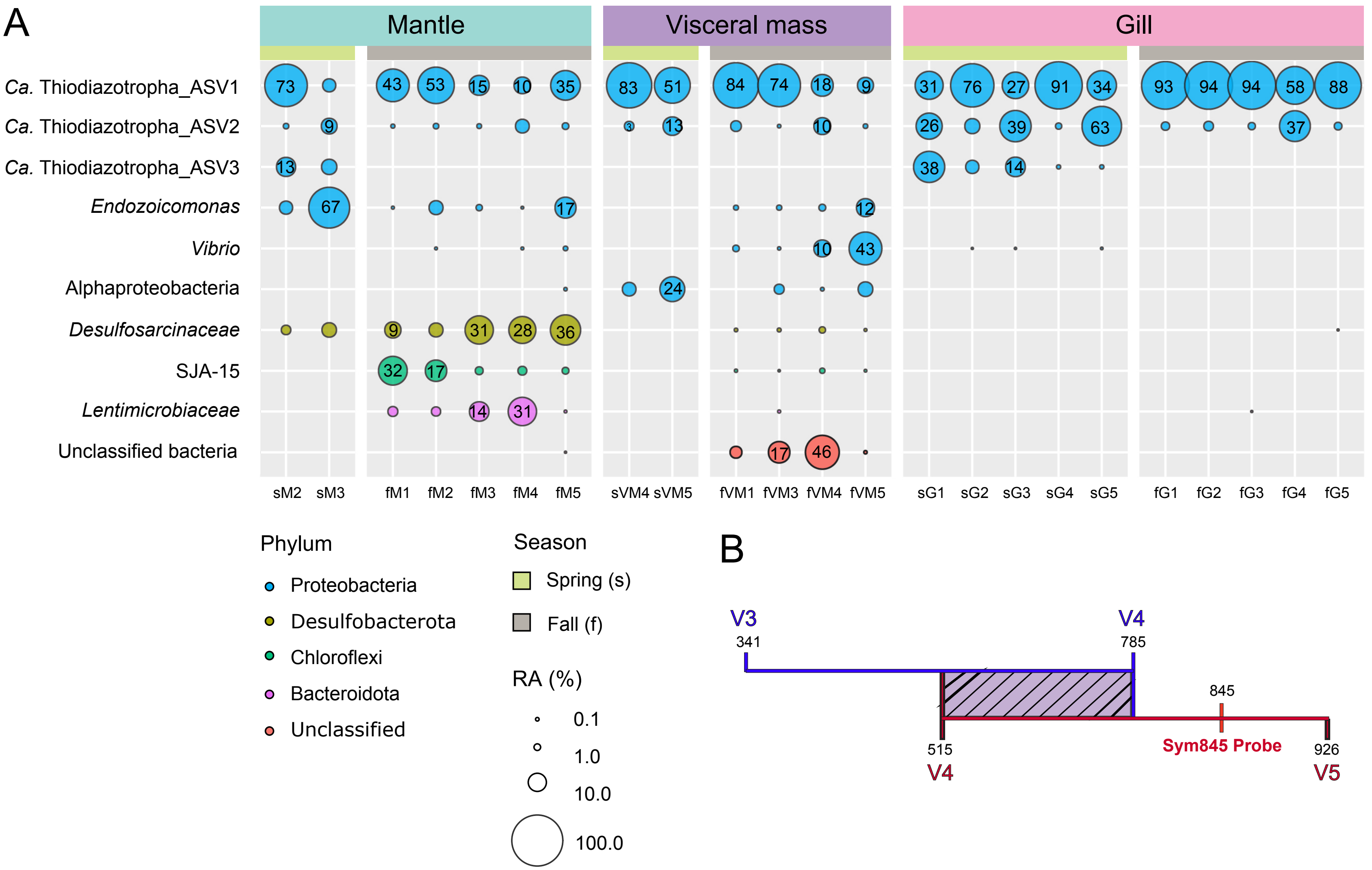
A
Mantle
Visceral mass
Gill
Ca. Thiodiazotropha_ASV1
Ca. Thiodiazotropha_ASV2
Ca. Thiodiazotropha_ASV3
Endozoicomonas
Vibrio
Alphaproteobacteria
Desulfosarcinaceae
SJA-15
Lentimicrobiaceae
Unclassified bacteria
sM2 sM3 fM1 fM2 fM3 fM4 fM5 sVM4 sVM5 fVM1 fVM3 fVM4 fVM5 sG1 sG2 sG3 sG4 sG5 fG1 fG2 fG3 fG4 fG5
73 43 53 15 10 35 83 51 84 74 18 9 31 76 27 91 34 93 94 94 58 88
9 3 13 10 26 39 63 37
13 38 14
67 17 12
10 43
24
9 31 28 36
32 17
14 31
17 46
Phylum
Proteobacteria
Desulfobacterota
Chloroflexi
Bacteroidota
Unclassified
Season
Spring (s)
Fall (f)
RA (%)
0.1
1.0
10.0
100.0
B
V3
341
V4
785
845
515
V4
Sym845 Probe
926
V5
