# Supplementary figures and images for: Sulfur-oxidizing symbionts colonize the digestive tract of their lucinid hosts
Source: ISME J. 2024 Oct 10;18(1):wrae200. doi: 10.1093/ismejo/wrae200 (PMC11549920; doi:10.1093/ismejo/wrae200)

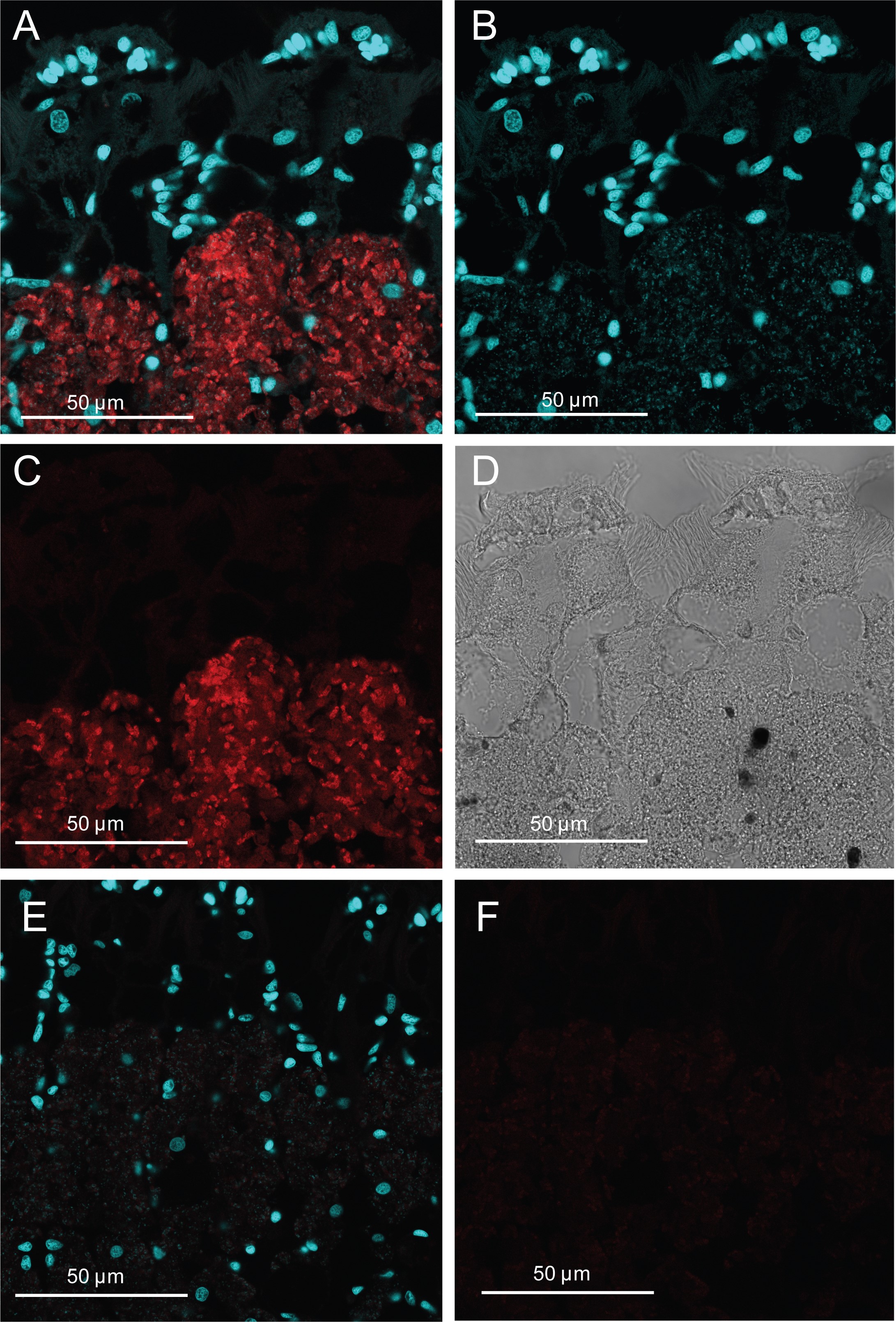

Supplement: FISH_revFigS3_wrae200 [file fish_revfigs3_wrae200.jpeg]

A

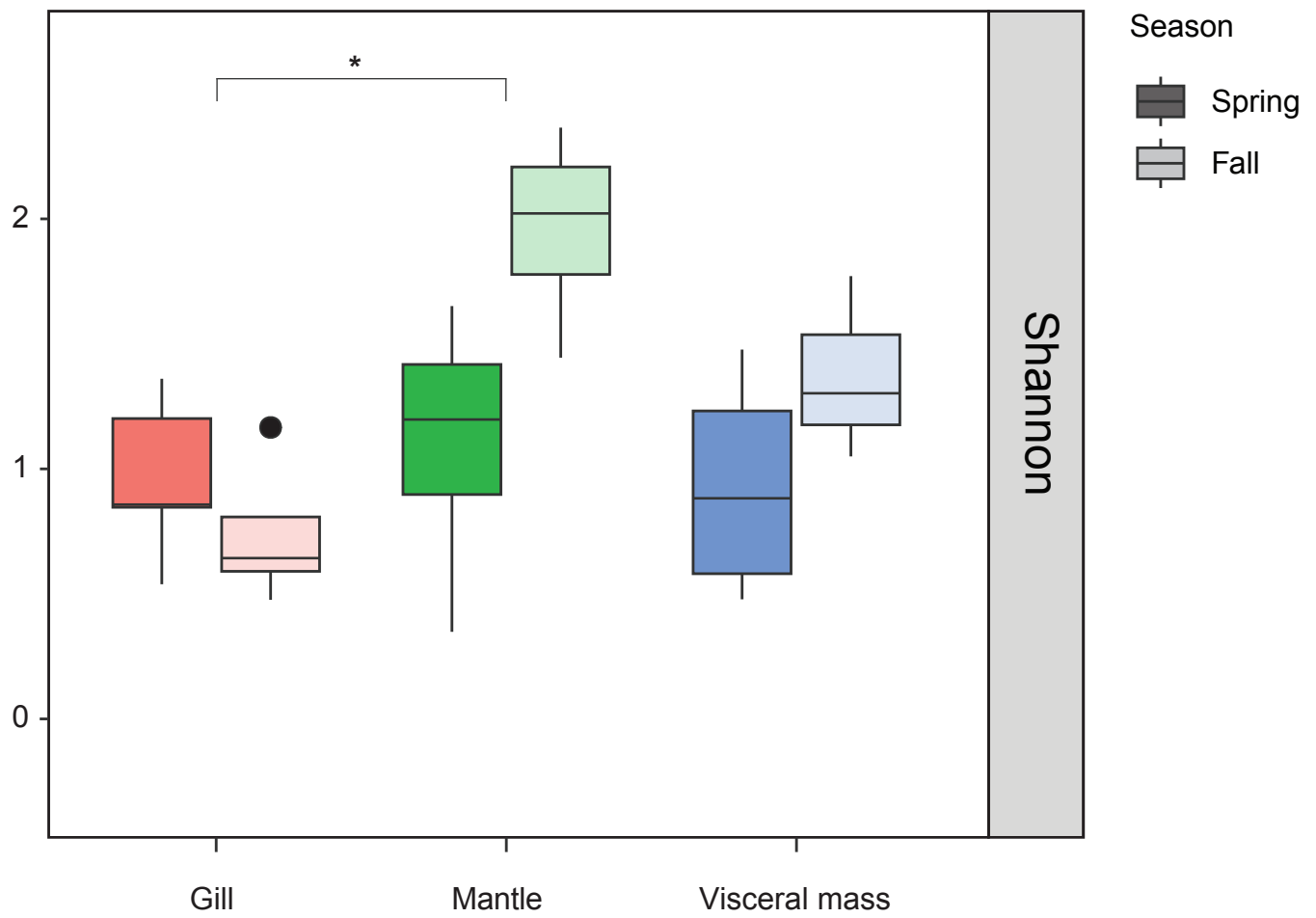

B

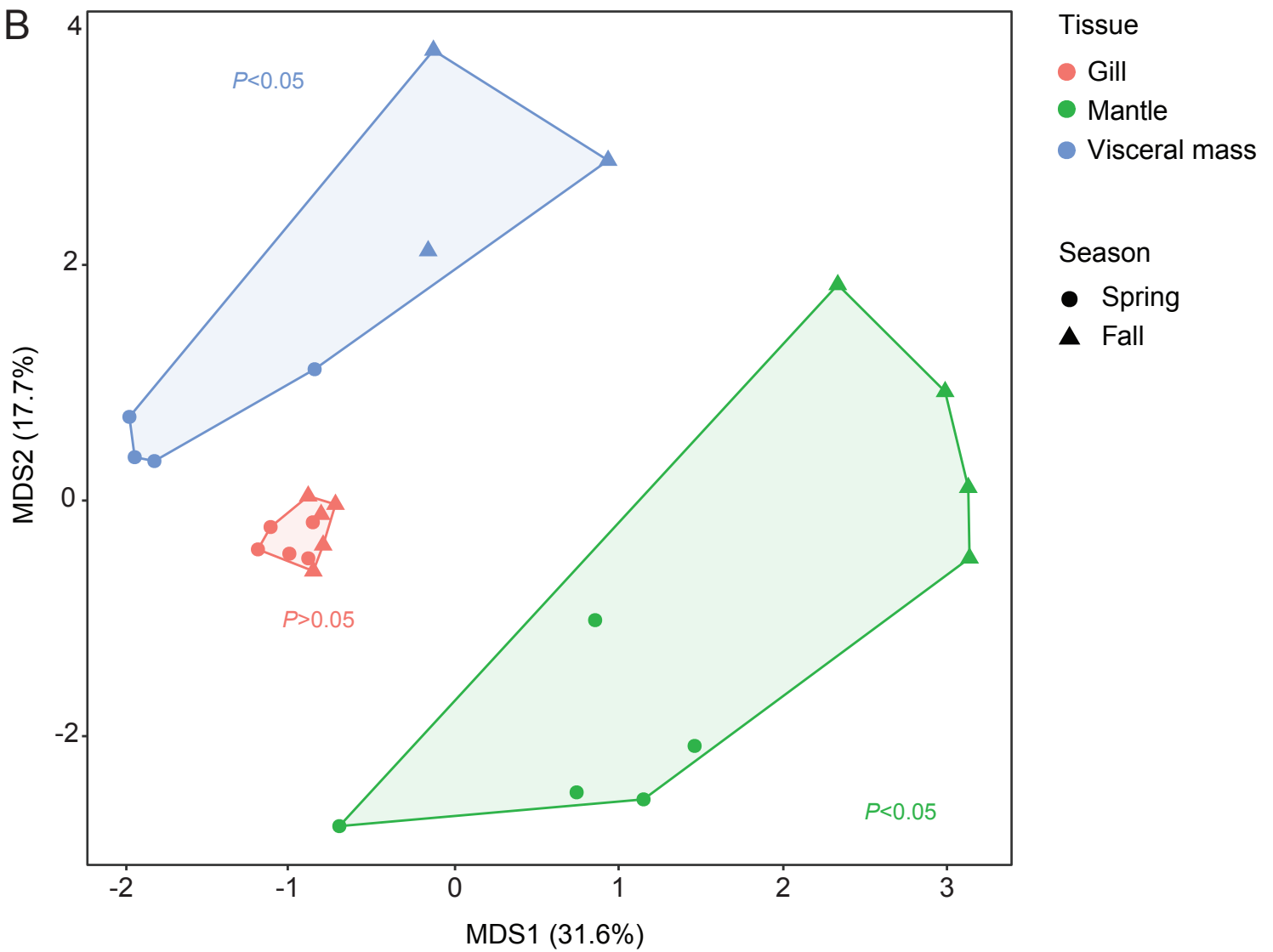

Supplement: FigS4AlphaBetaRev2_wrae200 [file figs4alphabetarev2_wrae200.pdf]

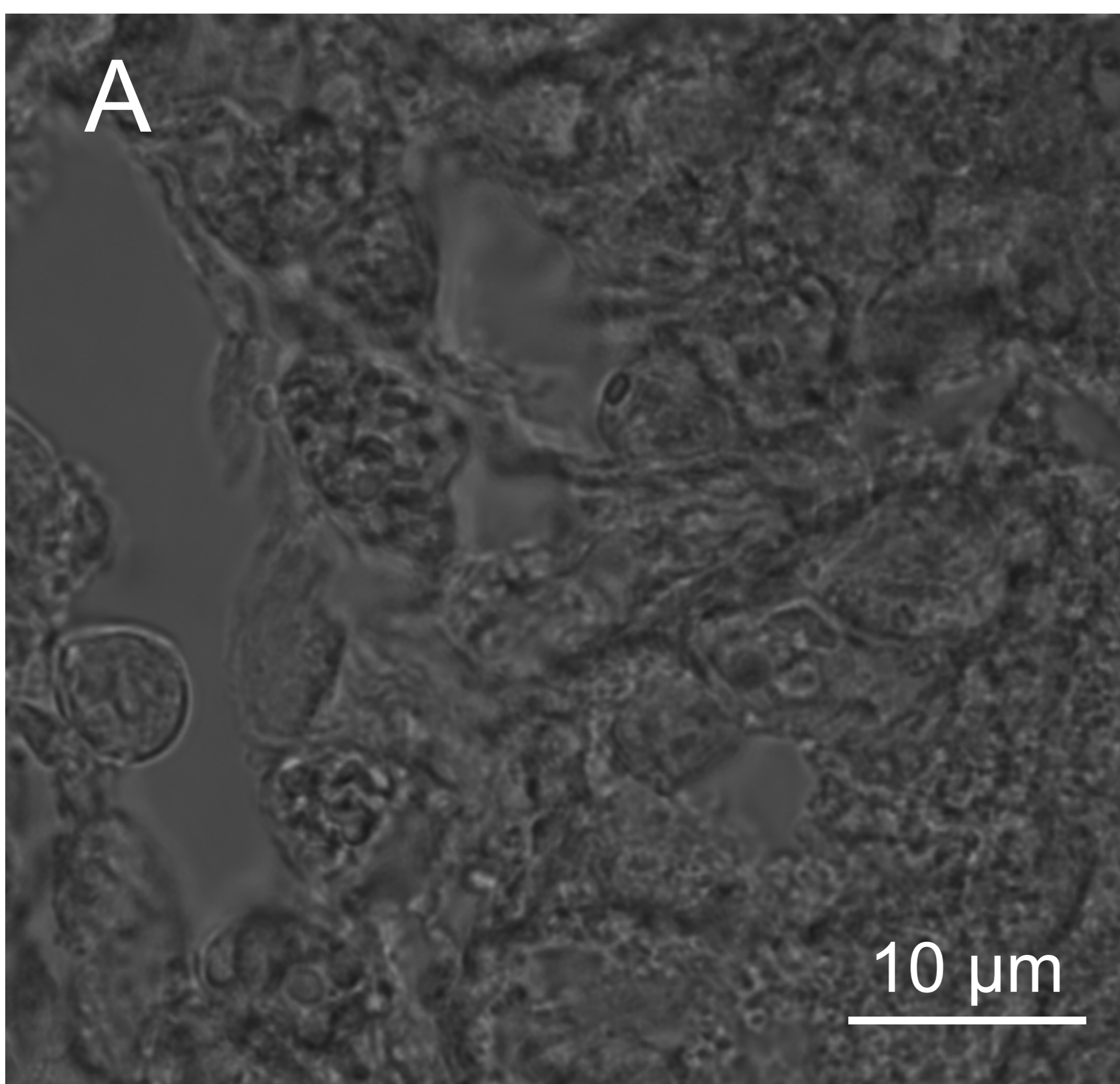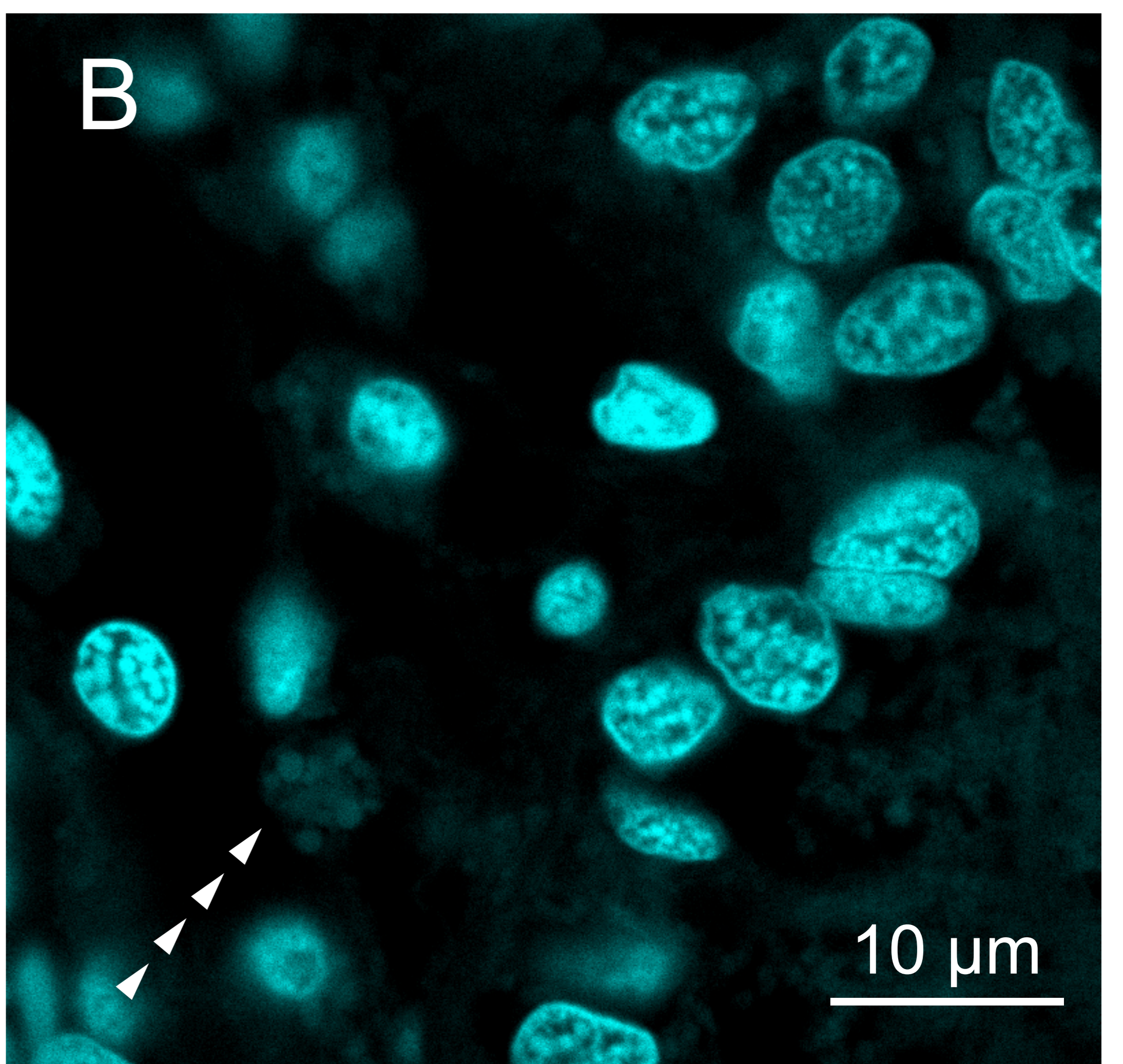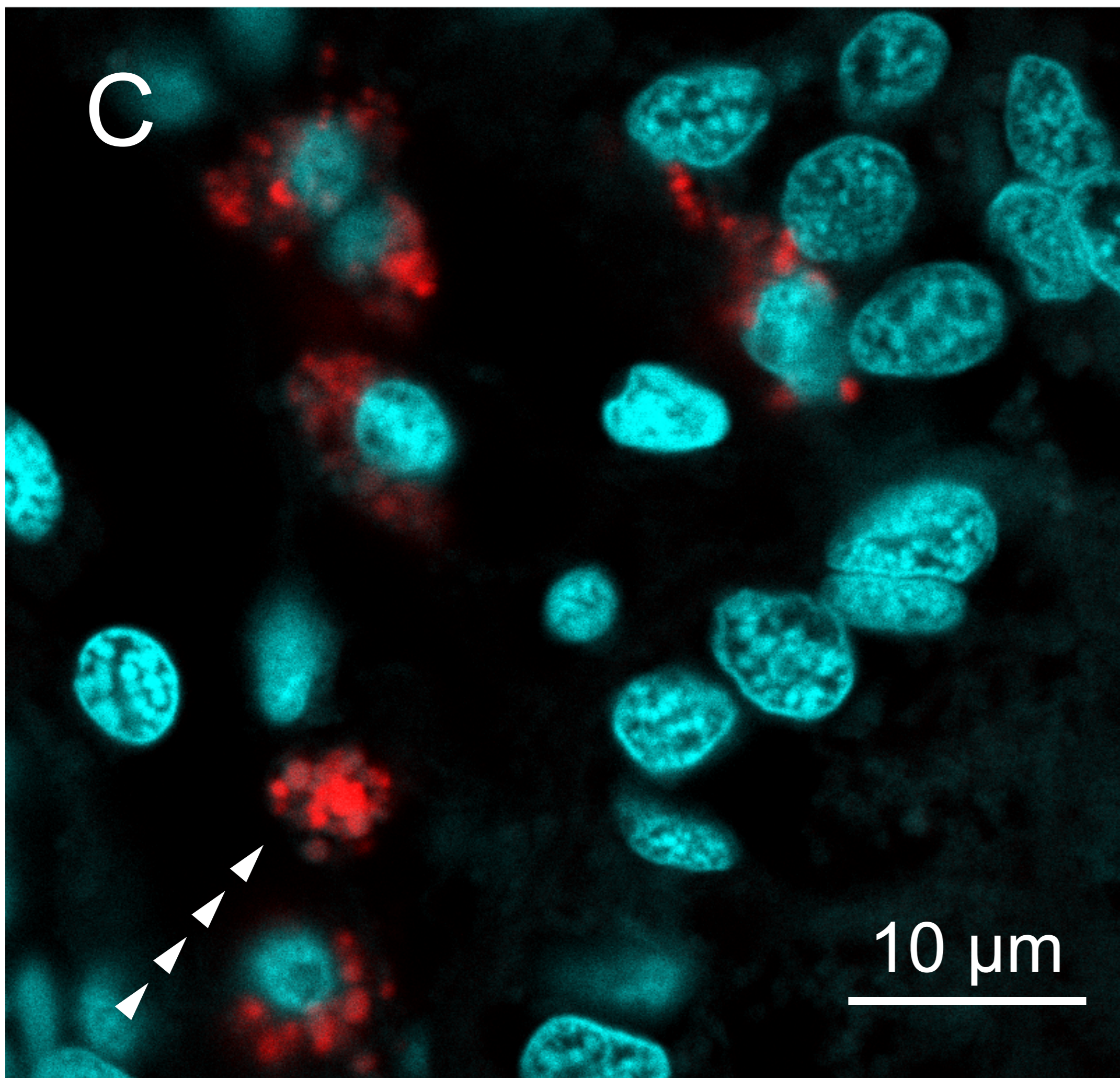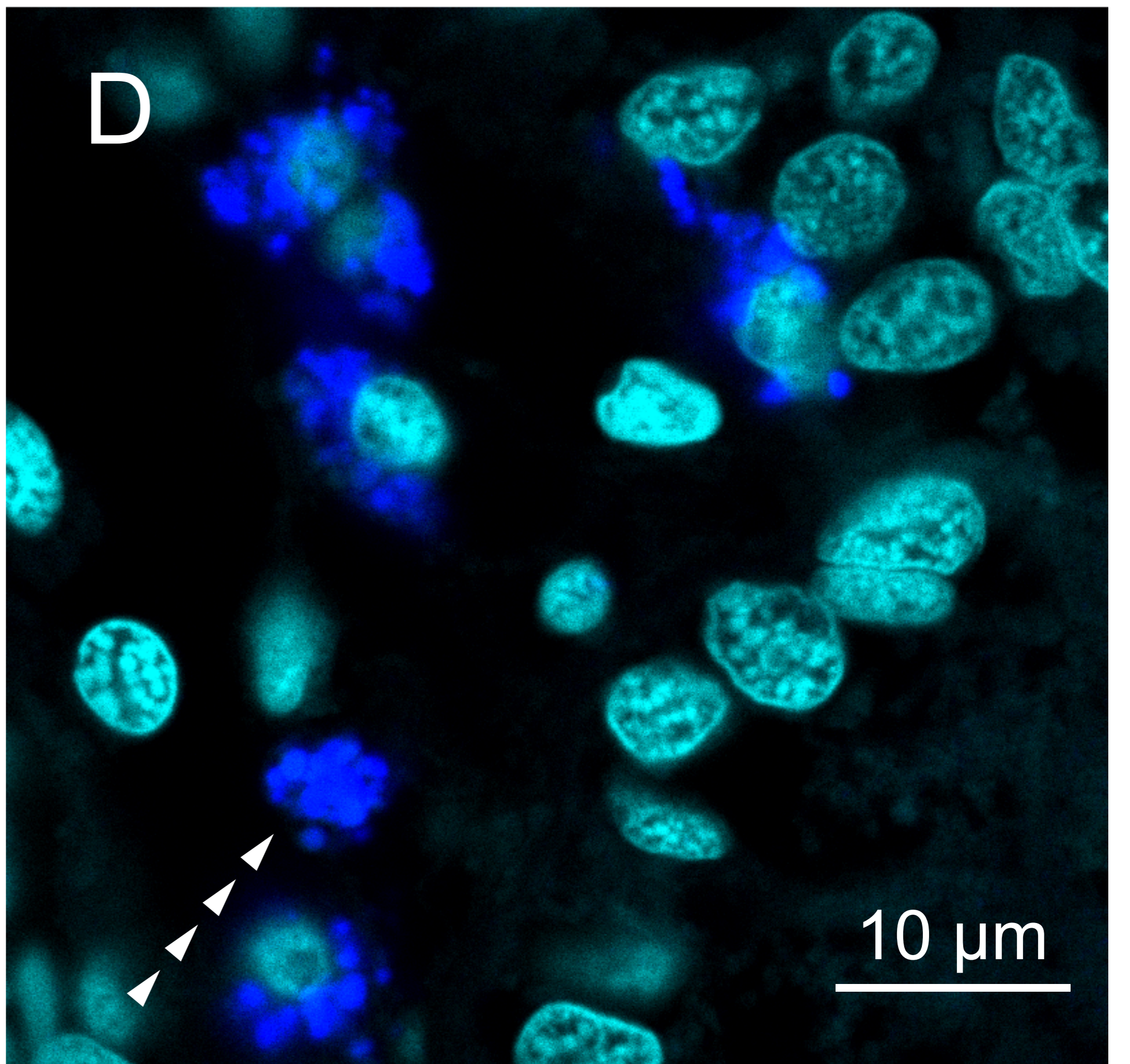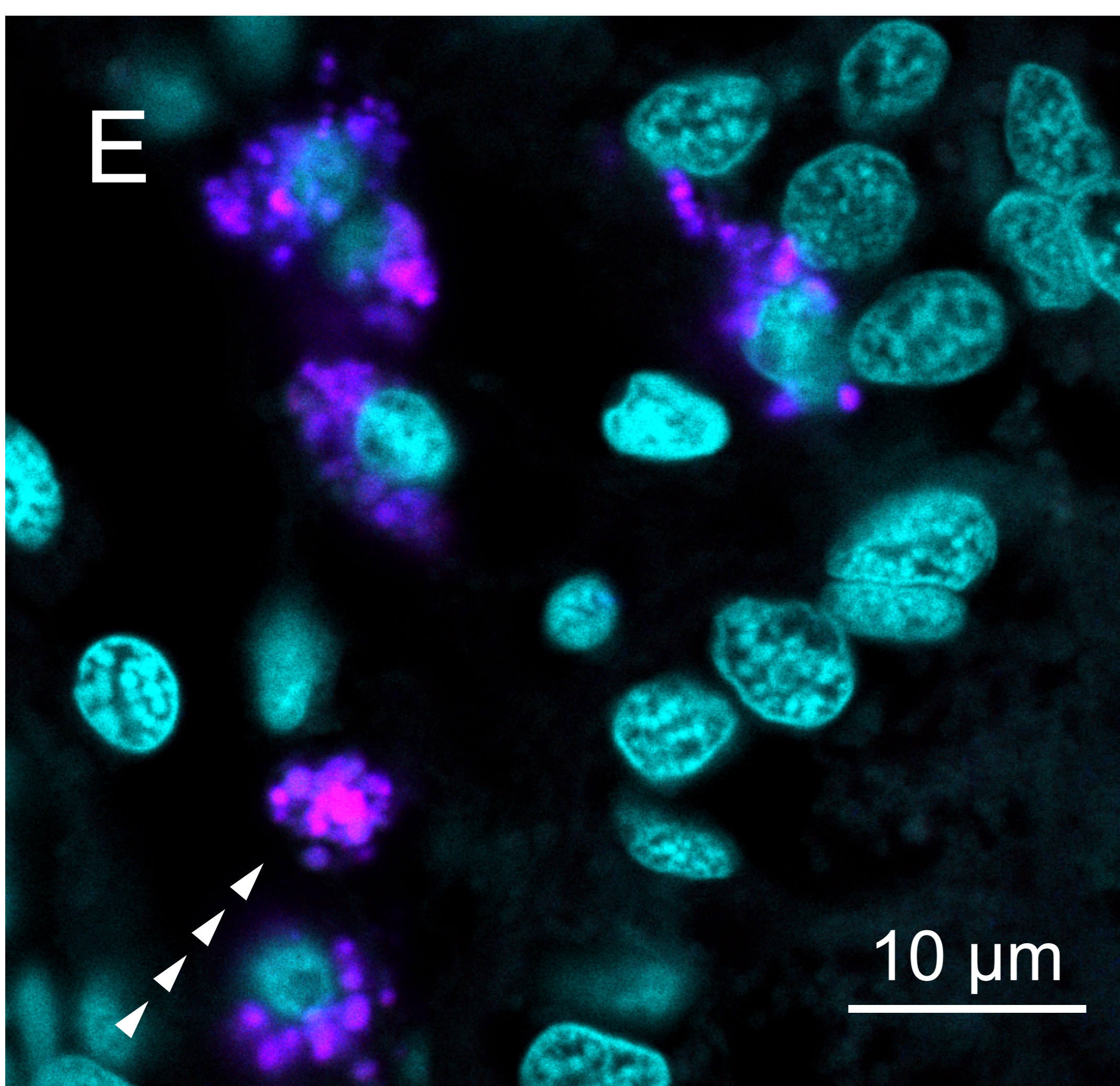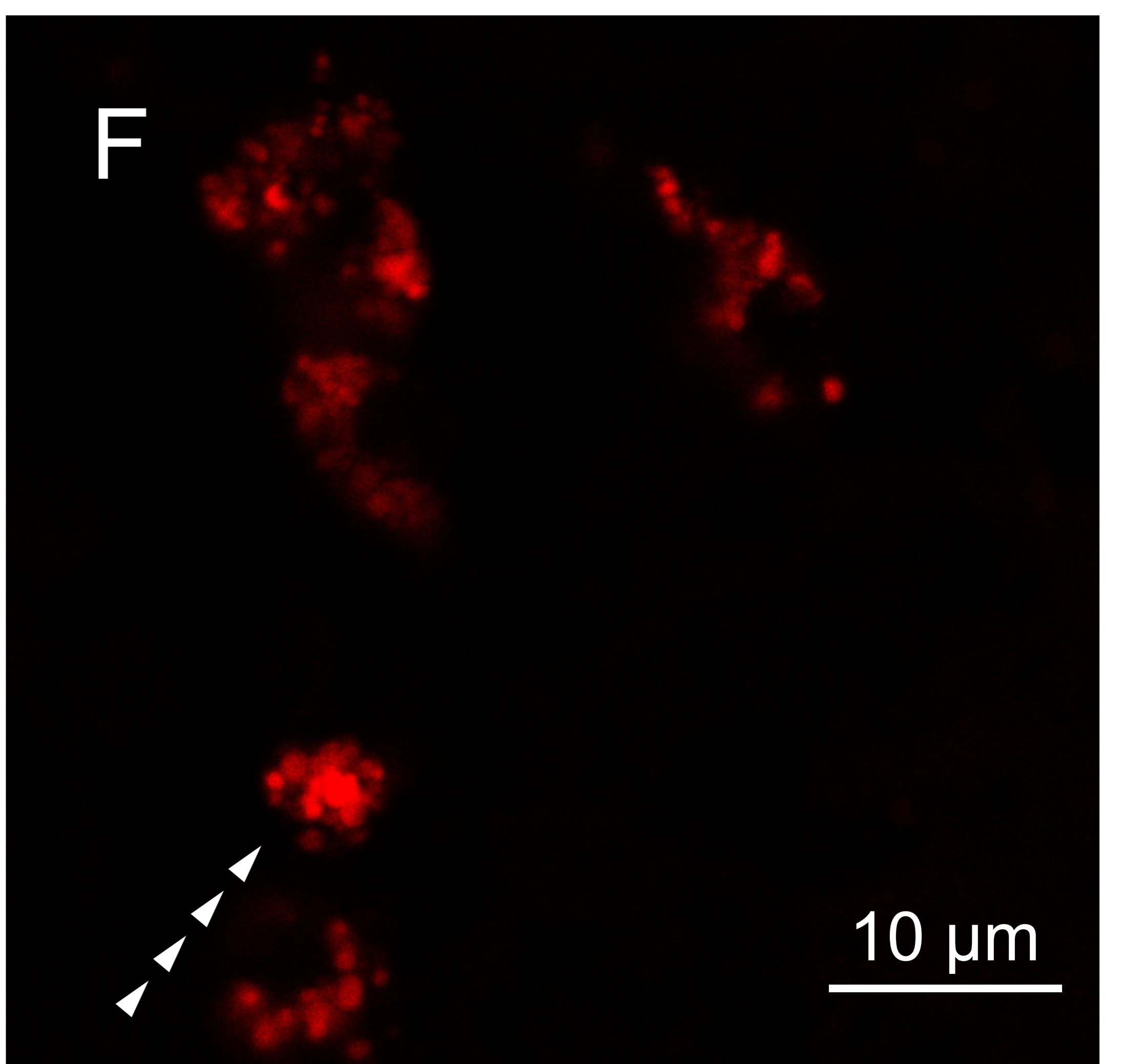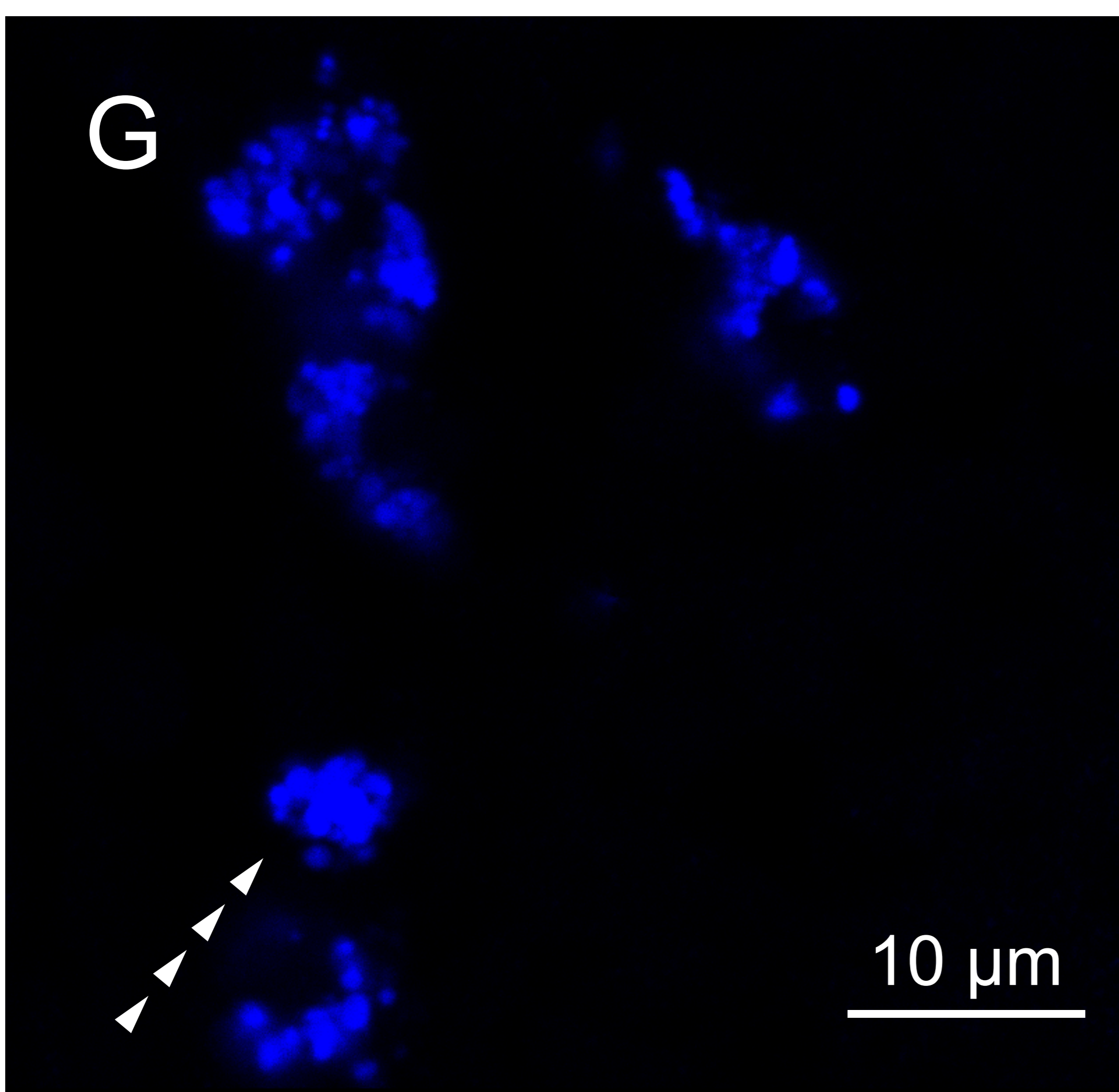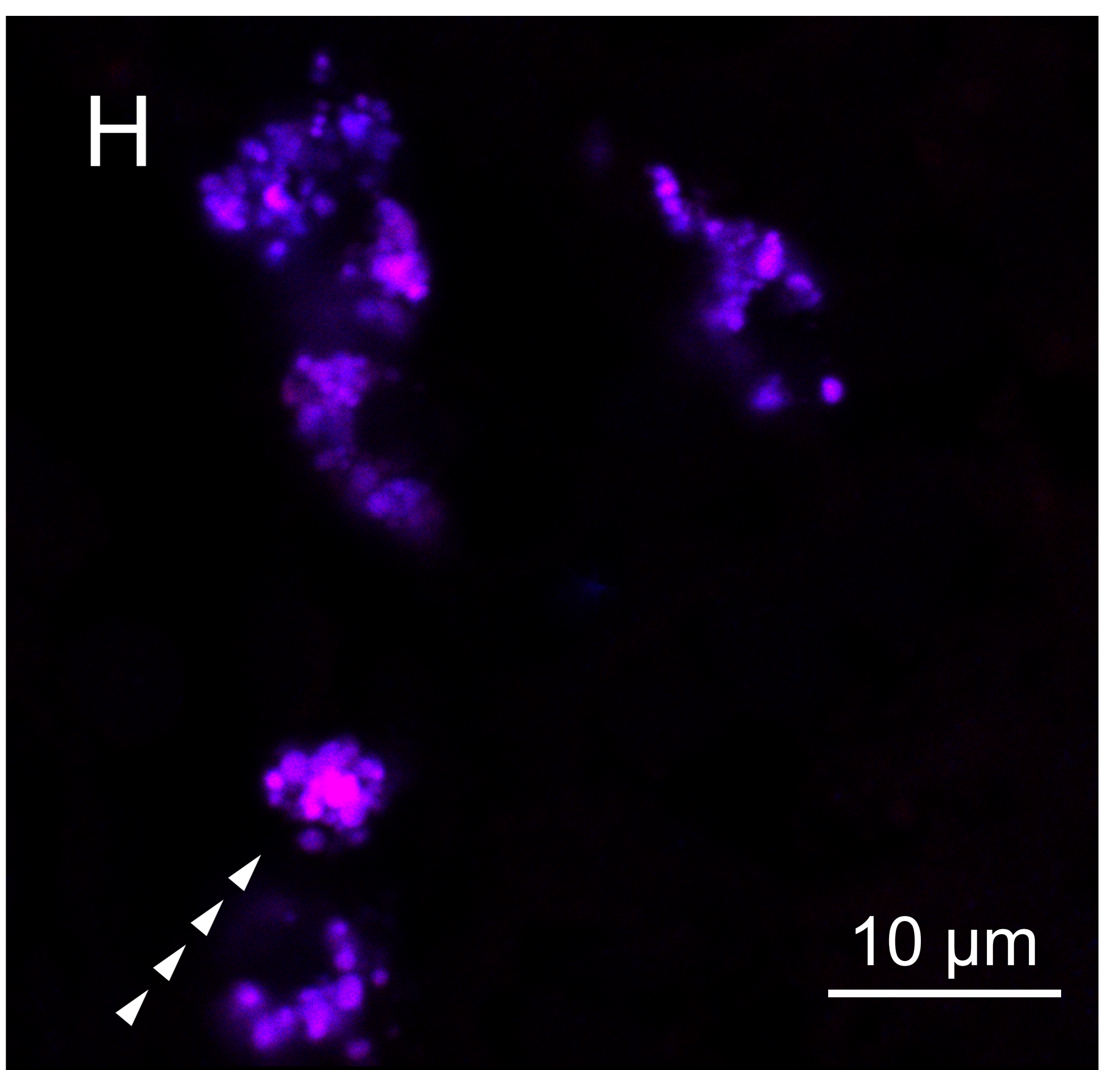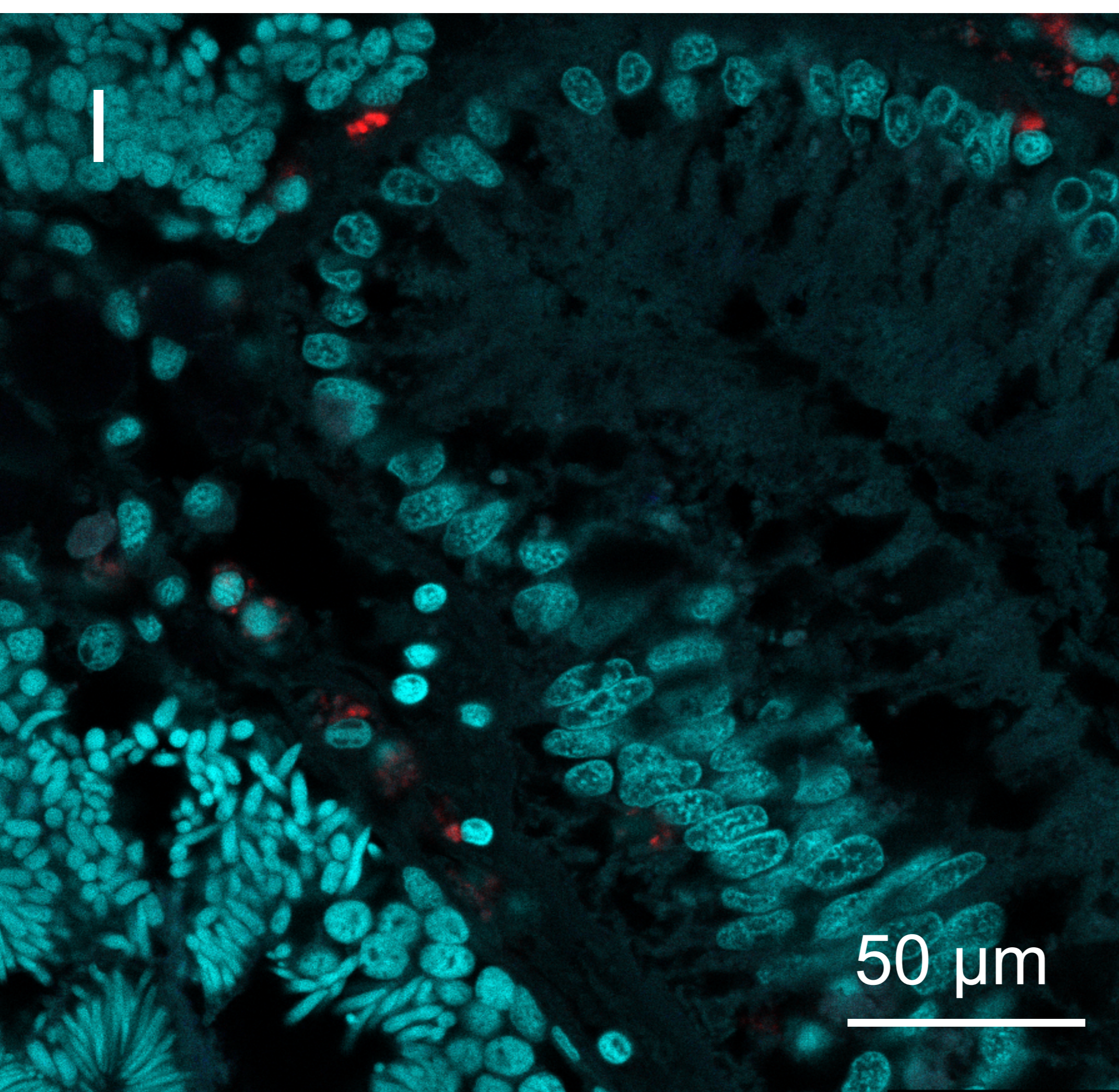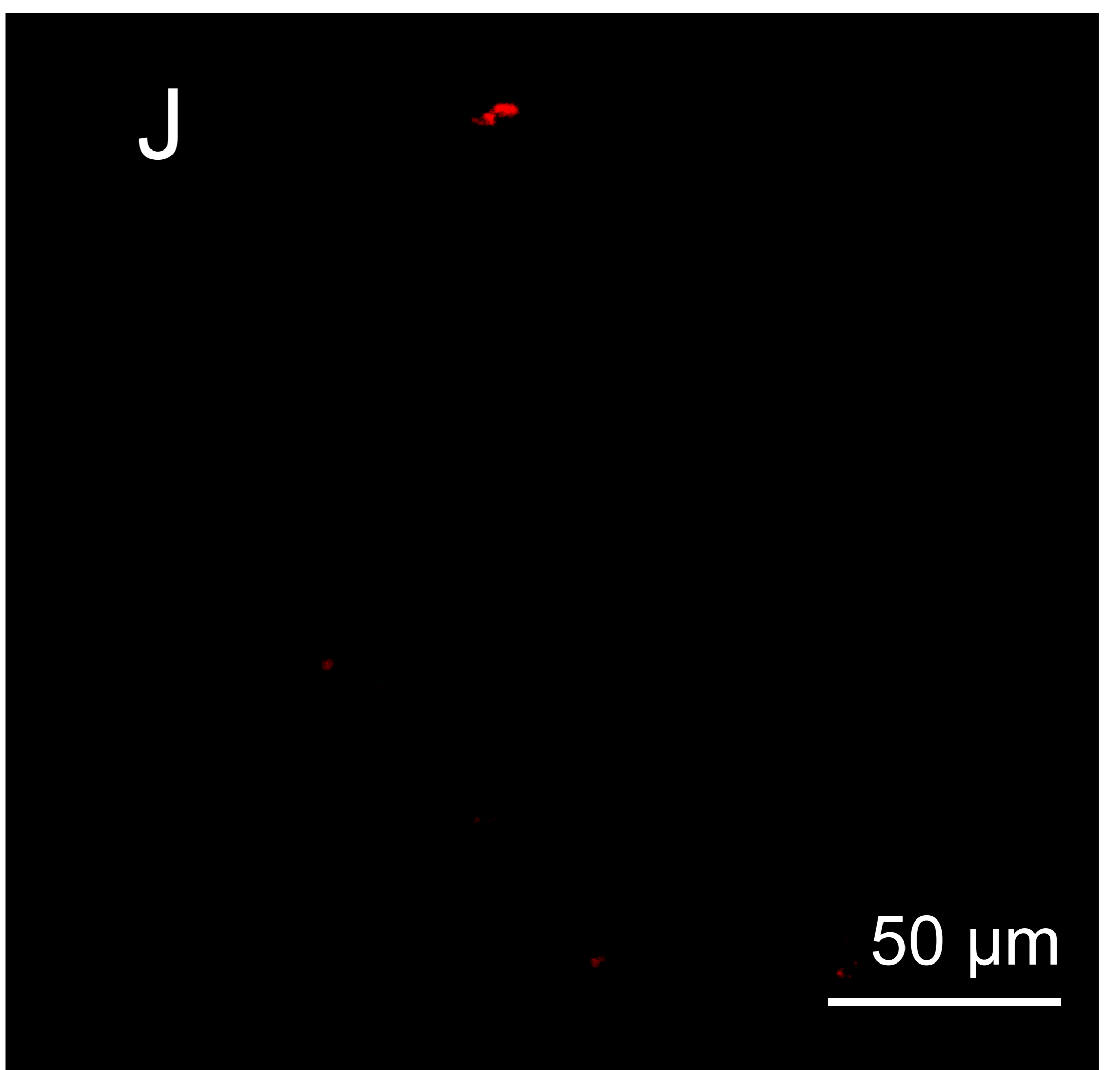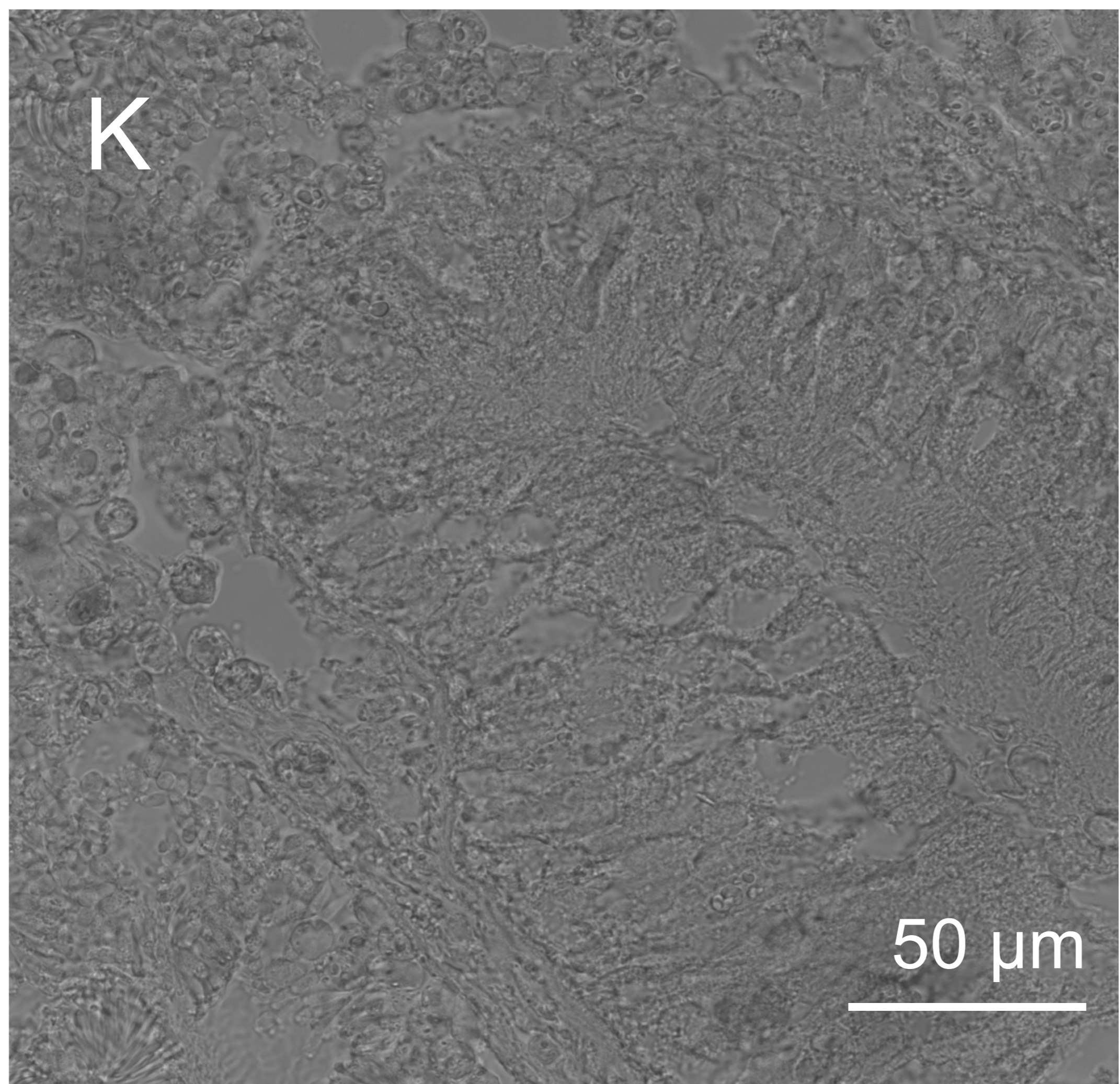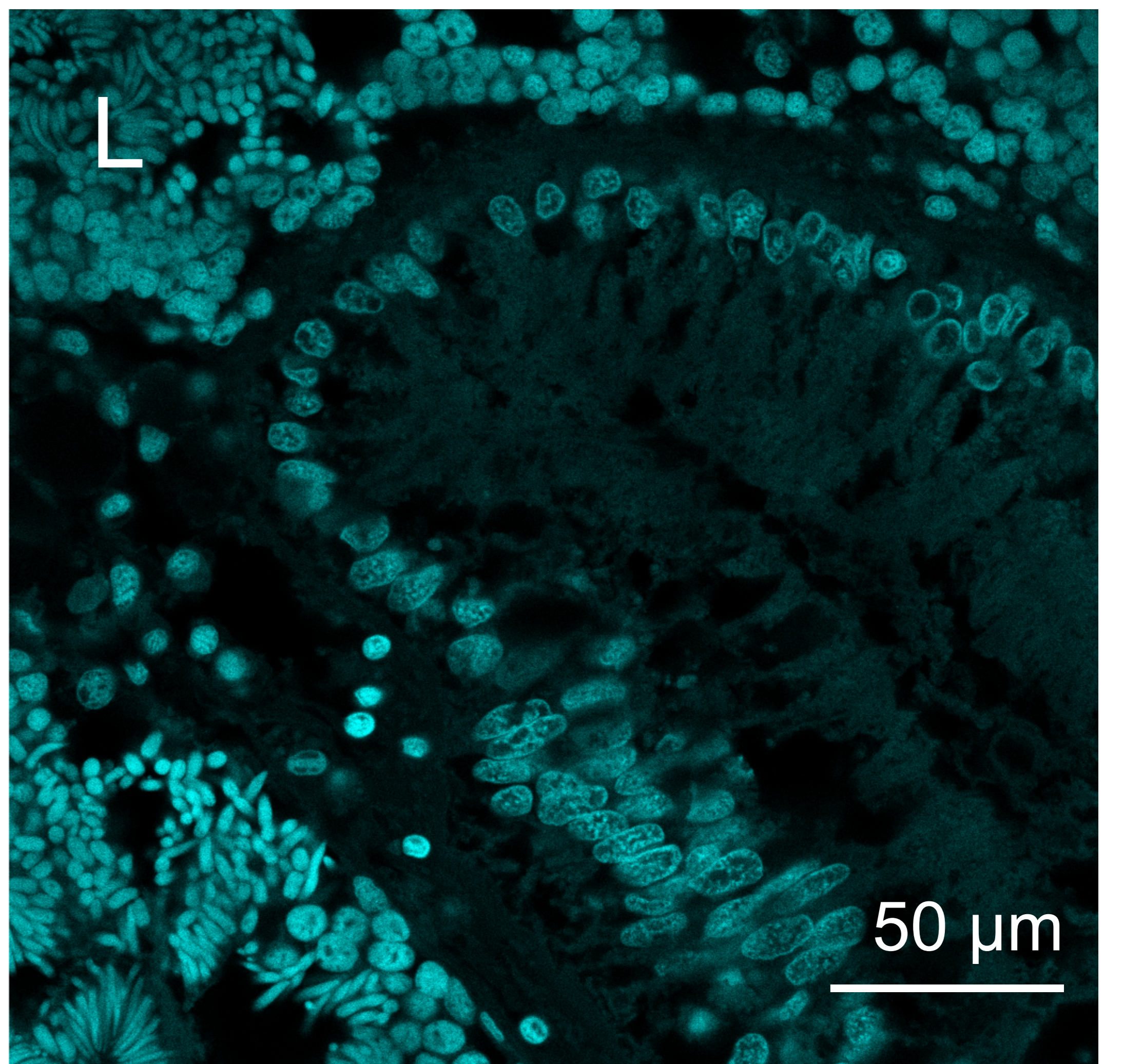

Supplement: FigS5_fresh_gut_wrae200 [file figs5_fresh_gut_wrae200.pdf]
