# Supplementary material for: Sulfur-oxidizing symbionts colonize the digestive tract of their lucinid hosts
Source: ISME J. 2024 Oct 10;18(1):wrae200. doi: 10.1093/ismejo/wrae200 (PMC11549920; doi:10.1093/ismejo/wrae200)
Supplement: Sup_SOX_col_no_images_wrae200 [file sup_sox_col_no_images_wrae200.docx]

**Sulfur-Oxidizing Symbionts Colonize the Digestive Tract of their Lucinid Hosts.**

Cristina M. Alcaraz, Joana Séneca, Martin Kunert, Christopher Pree, Marta Sudo, Jillian M. Petersen

Supplementary text

# Materials and Methods

## Sample collection

*Loripes orbiculatus* were collected from sediments near seagrass meadows (*Posidonia oceanica*) during fall (October 2023) and spring (May 2022) in the bay of Fetovaia (42°43.813’N 010°09.357’E) at approximately 7 m depth. The collection was conducted by the scuba team of the Elba Island station HYDRA Institute for Marine Sciences in Fetovaia, Italy, situated in the Mediterranean Sea. Clams and their natural sediments and seawater were subsequently transported to the University of Vienna for processing. Upon arrival, fresh clams for 16S rRNA gene sequencing and fluorescence *in situ* hybridization (FISH) experiments underwent immediate processing. Clams are opened with a scalpel, revealing the whole intact body. Precision scissors and tweezers are used to separate each tissue. There are three petri dishes per individual organ filled with filtered artificial seawater the organs are submerged in a rinsing series of three, to ensure as little contamination as possible. Clams were either dissected into four distinct tissues (foot, mantle, visceral mass, gills) and frozen in liquid nitrogen for downstream DNA extraction or were fixed whole in 4% paraformaldehyde (v/v) at 4°C overnight for subsequent FISH experiments.

## ‘Starvation’ Experiment

A subset of approximately 60 clams collected in Spring 2022 were exposed to starvation, where access to reduced sulfides for the chemoautotrophic symbionts was restricted. For this incubation, the sediments were washed and extensively rinsed with Red Sea Salt Filtered Artificial Seawater. The clams were housed under these nutrient-limited conditions in a plastic container in seawater at temperatures ranging from 20°C to 23°C for 12 to 14 months. Salinity was consistently maintained at 36 ppt, and seawater was aerated with an aquarium air stone and periodically replaced. A slow decline in the starving population was noticed at 12 months, with the death of multiple individuals on a weekly basis. When the number of deaths began to increase weekly, most of the population was dissected and prepared for FISH or DNA extractions. A few starving individuals survived for 14 months; these were dissected and preserved for FISH.

**FISH and confocal laser scanning microscopy**To visualize symbionts of the phylotype *Ca*. Thiodiazotropha within the host, we performed dope-FISH^1^ on multiple specimens of both fresh and starved *L*. *orbiculatus* (Fig 1, Fig S2, Fig S3). A formamide series from 0-70% in 5% increments was performed to identify the optimal 35% formamide hybridization conditions^2^. The symbiont specific probe Sym-845 (Table S1) was verified *in silico* using TestProbe 3.0 against the SILVA database r138.1 NR with zero mismatches. Out of 458,567 potential sequences in the database, the probe showed 212 perfect matches including our target organism as well as a limited number of non-target organisms (Table S2). Among the non-target organisms were several known chemosynthetic sulfur oxidizing symbionts of vestimentiferan tubeworms and ectosymbionts of marine nematode worms that are not reported to occur in lucinid clams. Additionally, several phototrophic purple sulfur bacteria from the Chromatiaceae were targeted. No members of any of these groups were detected in our samples through 16S rRNA gene sequencing (Table S2). For fixation, clams were opened, and the entire body was extracted from the shell. The dissected clams underwent fixation in a 4% paraformaldehyde pH 7.4, 0.01 M PBS solution overnight at 4°C. Subsequently, samples were washed in 0.01 M PBS for 10 minutes three times, followed by dehydration in an ethanol series at 30%, 50%, and 70% concentrations for 10 minutes each. The dehydrated whole-body tissues were embedded in 1% low melting agarose. An additional dehydration and paraffin wax infiltration was performed using the Automatic Tissue Processor Donatello (Diapath, Italy). Low melting temperature paraffin (ATS-200850, Sanova, Austria) was used for infiltration, followed by embedding in medium melting temperature paraffin (ATS-200856, Sanova, Austria). Embedded whole clams were sectioned at 5 μm using a Leica microtome (Leica, Germany) and then placed on SuperfrostPlus adhesion slides (Thermo Scientific, USA) in a 37°C water bath. The sections were left to dry horizontally face-up at room temperature overnight. Next, the sections were dewaxed in Roti-Histol (Carl Roth, Germany) for 10 minutes in three cycles, followed by two 10-minute washes with absolute ethanol. Lastly, sections were washed for 5 minutes, three times in 0.01 M PBS. Dewaxed sections were dried using compressed air and stored at 4°C. The universal bacterial probe mix (EUB-338 I-III) was used as a positive control labeled with Cy5 (false colored blue) and the symbiont probe was labeled with Cy3 (false colored red). Whole-body clam sections were incubated in 1:10 ratio of 50 ng μL^-1^ probe and hybridization buffer (900 mM NaCl, 20 mM Tris-HCL, 35% formamide (v/v), 10% SDS (v/v) at 46°C for 3 hours. After incubation, slides with whole-body sections were rinsed in room temperature (RT) wash buffer (70 mM NaCl, 20 mM Tris-HCL, 5 mM EDTA) and incubated in wash buffer in a 48°C water bath for 15 minutes. Slides were then transferred to ice-cold Milli-Q (MQ) H_2_O and quickly rinsed three times. Next, DAPI at 1 μg/mL was applied to each section for 15 minutes, then rinsed in MQ H_2_O and left to air dry in the dark. Slides were mounted with ProLong glass antifade mountant and left to set overnight. The Leica TCS SP8 X confocal laser scanning microscope was utilized to visualize the sections. Images were captured using a 93X Plan-Apochromat oil immersion objective (refractive index of glass slide, immersion oil, and antifade mounting medium: 1.52). Image acquisition and, if necessary, post-processing were carried out using the Leica software LASX (3.7.2.22383).

## DNA extraction, 16S rRNA gene amplicon sequencing and analysis

DNA extraction, 16S rRNA gene sequencing, and raw data processing was performed at the Joint Microbiome Facility of the Medical University of Vienna and the University of Vienna (project IDs JMF-2203-10 and JMF-2310-08) following a standard 2-step PCR protocol as described previously^6^. Briefly, DNA from the different tissues of fresh *L. orbiculatus* clams was extracted using the InnuPrep DNA Mini kit v. 2.0 (IST Innuscreen, Berlin, Germany) according to the manufacturer’s instructions. The V3-V4 hypervariable region of the 16S rRNA gene was amplified using primer pairs 341F (CCTACGGGNGGCWGCAG) and 785R (GACTACHVGGGTATCTAATCC)^7^. Because the SYM-845 FISH probe targets a region that is not fully within the V3-V4 region of the 16S rRNA gene, we further amplified and sequenced the V4-V5 region using primers 515F (GTGYCAGCMGCCGCGGTAA) AND 926R (CCGYCAATTYMTTTRAGTTT)^8^. Barcoded libraries were prepared for sequencing using the TruSeq Nano DNA Kit (Illumina) and sequenced on the Illumina MiSeq platform (V3 chemistry, 600 cycles). Amplicon pools were extracted from the raw sequencing data using the FASTQ workflow in BaseSpace (Illumina) with default parameters. Demultiplexing was performed with the python package demultiplex (Laros JFJ, github.com/jfjlaros/demultiplex) allowing one mismatch for barcodes and two mismatches for linkers and primers. Amplicon sequence variants (ASVs) were inferred using the DADA2 *R* package v1.30^9^ applying the recommended workflow^10^. FASTQ reads 1 and 2 were trimmed at 230 nt with allowed expected errors of 4 and 6, respectively. Sequence read pairs that could not be merged were concatenated. ASV sequences were subsequently classified using classifier implemented in DADA2 against the SILVA database SSU Ref NR 99 release 138.1^11,12^ using a confidence threshold of 0.5. Downstream analyses were performed using *R* v.4.3.2 and Bioconductor v3.16 packages SummarizedExperiment v1.32, SingleCellExperiment v1.24, TreeSummarizedExperiment v2.6 ^13^, phyloseq v1.44, ^14^ microbiome v1.22^15^, and microViz v0.10.5 ^16^. For both 16S rRNA datasets, (V3-V4 and V4-V5), we excluded ASVs without classification or classified as eukaryotes, mitochondria, chloroplasts, and known contaminants, as well as ASVs with an abundance < 0.01% per sample in at least 5 samples This resulted in about 9% total read loss but did not significantly change the relative abundance of the topmost abundant taxa, including *Ca.* Thiodiazotropha. For the V3-V4 16S rRNA gene dataset, we removed ASVs shorter than 350 bp since the expected amplicon size for this primer pair was about 427 bp. We used *R* package decontam v. 0.20^17^ to identify and further remove ASVs flagged as contaminants using a threshold of 0.5 against the extraction negative controls. After filtering, we retained all samples with more than 350 reads, which resulted in the exclusion of all foot samples. For the V4-V5 16S rRNA gene dataset, we detected a strong co-amplification of the host tissue in almost all samples. This included mitochondria and one specific Eukaryotic ASV which made up to 99% of all the reads in a sample. After removing these non-specific ASVs and filtering, we kept samples more than 150 reads, resulting in the loss of all foot samples and most of the spring mantle samples. In total, out of 40 initial samples, after filtering we retained 26 samples from the V3-V4 dataset (Fig. 1), and 23 from the V4-V5 dataset (Fig. S1). The relative abundance of the top 10 most abundant ASVs was visualized using *R* package ampvis2 v. 2.7.32^18^.

Alpha- and beta diversity metrics were estimated for the V3-V4 16S rRNA gene dataset. Briefly, alpha diversity (i.e. the Shannon diversity index) was calculated (Fig. S6 A) on rarified data (379 read pairs/sample). Differences in community structure were calculated on non-rarefied data by performing a PCoA (Fig. S6 B) with Aitchison distance. Significant differences between tissues, as well as differences between seasons within the same tissue were tested with a PERMANOVA using the Aitchison distance. Differences in group dispersions (i.e different tissues) were assessed by an analysis of multivariate homogeneity (PERMDIST).

**Digital polymerase chain reaction (dPCR)**

DNA extracted from *L. orbiculatus* tissues (foot, mantle, visceral mass, and gills) from the fall, October 2023 and from 12-month starved tissues collected in May 2022 was analyzed using the QIAcuity One dPCR system (Qiagen). The primer pair soxB_1_f (5’-ACCGATACCCATGCACAACTCA-3’) and soxB_1_r (5’-TTGAGCAGATTATCGCCCACCA-3’) were designed using Primer3Plus^x^ to target the *soxB* gene of *Ca.* Thiodiazotropha weberae and *Ca.* Thiodiazotropha lotti, producing amplicons of 113 bp size. Amplification of the *soxB* gene was carried out using the QIAcuity EG PCR Kit (Qiagen, 250111) with EvaGreen PCR Master Mix following the manufacturer’s instructions. Briefly, for fall samples a final reaction volume of 12 µl was prepared with a primer concentration of 0.4 µM, using 2 µl of a 2.5 ng/µl DNA stock, diluted 1:1,000 for foot, mantle, and visceral mass and 1:100,00 for gills. The reactions were run on the QIAcuity One machine (Qiagen), using a 24-well plate with 8500 partitions per well QIAcuity Nanoplate 8.5k 24-well (Qiagen, 250011) and analyzed with the QIAcuity software suite v2.5.0.1. (Qiagen). For starved samples a 40 µl reaction volume was prepared with the same primer concentration, using 8 µl of a 2.5 ng/µl stock of DNA that was diluted 1:1,000 for foot, mantle, and visceral mass and 1:100,00 for gills. These reactions were also run on the QIAcuity One machine (Qiagen), using a 24-well plate with 26000 partitions per wel lQIAcuity Nanoplate 26k 24-well (Qiagen, 250001), followed by analysis with the same software. The following protocol was used for both runs: initial heat activation denaturation step at 95℃ for 5 minutes followed by 35 cycles of denaturation at 95℃ for 45 seconds, annealing at 63℃ for 30 seconds and extension at 72℃ for 1 minute, and finally a cooling step at 40℃ for 5 minutes. Controls for both runs included a non-template control of 16S amplicons from the 341F and 785R primer pair, a positive control of the *soxB* amplicons from the *soxB*_1_f/*soxB*_1_r primer pair and a negative control , using sterile water in place of DNA to the reaction. Standard PCR was also performed using the *soxB*_1_f/*soxB*_1_r primer pair on sample DNA. The PCR reaction volume was set up according to the manufacturer’s instructions with 5 ng template DNA, 5 µl 10X DreamTaq Green Buffer (Thermo Fisher, B71), 0.5 µL DreamTaq DNA Polymerase (Thermo Fisher, EP0703), 0.4 µM of each primer and filled up with Nuclease free water to a final volume of 50 µL. The PCR reaction was performed in a T100 Thermal Cycler (BioRad) using the following parameters: DNA initial denaturation at 95℃ for 5 minutes,followed by 40 cycles of denaturation at 95℃ for 30 seconds and a combined annealing and extension step at 60℃ for 1 minute, finally a stabilization step at 4°C for 5 minutes, followed by 90°C for 5 minutes. All steps had a temperature ramp of 2°C/s. The PCR product was sent for sanger sequencing and the resulting sequence blasted against the soxB gene in *L. orbiculatus*, the sequence aligned with 100% identity at the 146 - 205 bp position.To calculate the original sample’s copies per µL the following calculation was used: cp/µL value from the machine * (reaction volume [µL] ÷ template volume [µL]) * dilution factor and converted then to copies/ng of DNA using the total amount of DNA used in the reaction (Fig. 1B).

Foot

Mid-gut

B

# Supplementary Results and Discussion

Gut microbiomes of lucinid clams (represented by the visceral mass samples) showed distinct diversity patterns compared to those typical of filter-feeding bivalves. To begin with, in lucinids, most taxa were detected in all organs. This contrasts with e.g., well-studied zebra mussels where each organ (gut, mantle, gills) tends to host distinct taxa^19,20^. Our seasonal sampling also allows us to speculate about seasonality and fluctuations in the microbiome of lucinid clams, with the limitation that each season was only sampled once. However, at a broad level, seasonal fluctuations in the microbiome of filter-feeding oysters are common^21^, whereas the diversity of lucinid clam microbiomes was not obviously different in the two seasons sampled.

Bivalves not known to host chemosynthetic symbionts such as the well-studied oyster genus *Crassostrea* commonly host a highly diverse array of bacteria in the gills as well as the gut, with a notable plasticity in the gut microbiome^22^. Moreover, the microbiome associated with oysters and mussels without chemosynthetic symbionts varies according to host species diversity, host organ, geographical location, and environmental conditions^19,20,23^. Although studies have shown evidence of a putative core gut microbiome, these microbiomes are predominantly composed of transient or opportunistic bacteria acquired during filter-feeding^24^. In contrast, the lucinid gill microbiota was consistently dominated by a single gammaproteobacterial species across hosts from three distinct genera^25–27^. The role of resident microbiota in promoting host health and preventing pathogen colonization is well-established^28^, nevertheless, environmental stressors such as rising temperatures can induce shifts in microbiome composition and destabilization of a healthy microbiota in many non-symbiotic bivalves, including oysters^29^. Given the substantial reliance of lucinids on their endosymbionts for nutrition, it is plausible that this symbiotic relationship may confer a degree of resilience to pathogens, as was postulated for the sulfur-oxidizing symbionts of deep-sea *Bathymodiolus* mussels^30^. The consistent presence of a dense population of beneficial symbionts in all adults collected from the field, as seen in lucinids, suggests a stable and co-evolved microbiome that could potentially outcompete or inhibit the establishment of pathogenic microorganisms. Additionally, the specialized nature of the symbiotic association in lucinids may provide a distinct microenvironment within the host, limiting opportunities for pathogen colonization. However, further research is needed to explore the mechanisms underlying the potential pathogen resistance conferred by symbiosis in lucinids and to evaluate how environmental stressors, such as changing temperatures or pollution, may impact this relationship, the resilience of lucinid populations, and the diversity of other microbial associates.

In deep-sea *Bathymodiolus* mussels, which also acquire their symbionts from the surrounding environment, intracellular symbionts colonize the epithelia of several organs in juvenile mussels before becoming restricted to the gills later in development^31^. However in another study examining early symbiont colonization in Mytilidae mussels, correlative imaging analyses showed fully colonized gills, while mantle tissue were still in the process of being colonized^32^. Given that in bivalves, the epithelia lining the gill filaments develop from mantle and visceral epithelia^33,34^, it is conceivable that the symbionts present in these pre-gill epithelial cells may serve as a source population for symbionts in the gill. Moreover, dividing bacteria were observed in bacteriocytes in non-gill tissues, suggesting the capability for these bacteria to proliferate in these tissues^33^. Experimental data on symbiont activity, cell division and expression will help in future to understand the role of the extra-gill symbiont populations for the holobiont.

**Additional References**

1. Stoecker, K., Dorninger, C., Daims, H. & Wagner, M. Double labeling of oligonucleotide probes for fluorescence in situ hybridization (DOPE-FISH) improves signal intensity and increases rRNA accessibility. *Appl. Environ. Microbiol.* **76**, 3, 922-6 (2010)

2. Martin, B. C. *et al.* Cutting out the middle clam: lucinid endosymbiotic bacteria are also associated with seagrass roots worldwide. *ISME J* **14**, 2901–2905 (2020).

3. Ri, A. *et al.* Combination of 16S rRNA-targeted oligonucleotide probes with flow cytometry for analyzing mixed microbial populations. *Appl. Environ. Microbiol.* **56**, 6, 1919-25(1990).

4. Loy, A. *et al.* Oligonucleotide microarray for 16S rRNA gene-based detection of all recognized lineages of sulfate-reducing prokaryotes in the environment. *Appl. Environ. Microbiol.* **68**, 10, 5064-81 (2002).

5. Wallner, G. *et al*. Optimizing fluorescent in situ hybridization with rRNA-targeted oligonucleotide probes for flow cytometric identification of microorganisms. *Cytometry* **14**, 2, 136-43(1993).

6. P, P. *et al.* An economical and flexible dual barcoding, two-step PCR approach for highly multiplexed amplicon sequencing. *Front. Microbiol.* **12**, 669776 (2021).

7. Klindworth, A. *et al.* Evaluation of general 16S ribosomal RNA gene PCR primers for classical and next-generation sequencing-based diversity studies. *Nucleic Acids Res.* **41**, 1,e1 (2013).

8. Ae, P., Dm, N. & Ja, F. Every base matters: assessing small subunit rRNA primers for marine microbiomes with mock communities, time series and global field samples. *Environ. Microbiol.* **18**, 1403-1414 (2016).

9. Bj, C. *et al.* DADA2: High-resolution sample inference from Illumina amplicon data. *Nat. methods* **13**, 581-583 (2016).

10. Callahan, B. J., Sankaran, K., Fukuyama, J. A., McMurdie, P. J. & Holmes, S. P. Bioconductor workflow for microbiome data analysis: from raw reads to community analyses. *F1000Research* **5**, 1492 (2016).

11. Silva 138.1 prokaryotic SSU taxonomic training data formatted for DADA2. doi:10.5281/zenodo.4587955.

12. C, Q. *et al.* The SILVA ribosomal RNA gene database project: improved data processing and web-based tools. *Nucleic acids Res.* **41**, D590-6 (2013).

13. Huang, R. *et al.* TreeSummarizedExperiment: a S4 class for data with hierarchical structure. *F1000Research* **9**, 1246 (2021).

14. McMurdie, P. J. & Holmes, S. phyloseq: An R package for reproducible interactive analysis and graphics of microbiome census data. *PLOS ONE* **8**, e61217 (2013).

15. Microbiome@GitHub. https://microbiome.github.io/.

16. Barnett, D. J. m, Arts, I. C. w & Penders, J. microViz: an R package for microbiome data visualization and statistics. *Journal of Open Source Software* **6**, 63, 3201 (2021).

17. Davis, N. M., Proctor, D. M., Holmes, S. P., Relman, D. A. & Callahan, B. J. Simple statistical identification and removal of contaminant sequences in marker-gene and metagenomics data. *bioRxiv.* **6**, 1, 226 (2018) doi:10.1101/221499.

18. GitHub - KasperSkytte/ampvis2: Tools for visualising microbial community amplicon data. *GitHub* https://github.com/KasperSkytte/ampvis2.

19. Winters, A. D., Marsh, T. L. & Faisal, M. Heterogeneity of bacterial communities within the zebra mussel *(Dreissena polymorpha)* in the Laurentian Great Lakes Basin. *Journ.Great Lakes Res.***37**, 2, 318–324 (2011).

20. Musella, M. *et al.* Tissue-scale microbiota of the Mediterranean mussel *(Mytilus galloprovincialis)* and its relationship with the environment. *Sci. Total Environ.* **717**, 137209 (2020).

21. Pierce, M. L. & Ward, J. E. Gut microbiomes of the eastern oyster (*Crassostrea virginica*) and the blue mussel (*Mytilus edulis*): temporal variation and the influence of marine aggregate-associated microbial communities. *mSphere* **4**, 10.1128/msphere.00730-19 (2019).

22. Stevick, R. J., Post, A. F. & Gómez-Chiarri, M. Functional plasticity in oyster gut microbiomes along a eutrophication gradient in an urbanized estuary. *anim. microbiome* **3**, 1–5 (2021).

23. Hernández‐Zárate, G. & Olmos‐Soto, J. Identification of bacterial diversity in the oyster *Crassostrea gigas* by fluorescent in situ hybridization and polymerase chain reaction. *J. Appl. Microbiol.* **100**, 664–672 (2006).

24. Akter, S. *et al.* Host species and environment shape the gut microbiota of cohabiting marine bivalves. *Microb. Ecol.* **86**, 1755-1772 (2023).

25. Lim, S. J. *et al.* Extensive thioautotrophic gill endosymbiont diversity within a single *Ctena orbiculata* (Bivalvia: Lucinidae) population and implications for defining host-symbiont specificity and species recognition. *mSystems* 4, 10.1128/msystems.00280-19 (2019)

26. Lim, S. J. *et al.* Gill microbiome structure and function in the chemosymbiotic coastal lucinid *Stewartia floridana*. *FEMS Microbiol. Ecol.* **97**, 4 fiab042 (2021).

27. Lim, S. J. *et al.* Taxonomic and functional heterogeneity of the gill microbiome in a symbiotic coastal mangrove lucinid species. *ISME J* **13**, 902–920 (2019).

28. Chiu, L. *et al.* Protective Microbiota: from localized to long-reaching co-immunity. *Front. Immunol.* **8**, 1678 (2017).

29. Masanja, F. *et al.* Bivalves and microbes: a mini-review of their relationship and potential implications for human health in a rapidly warming ocean. *Front. Mar. Sci.* **10**, 1182438 (2023).

30. Sayavedra, L. *et al.* Abundant toxin-related genes in the genomes of beneficial symbionts from deep-sea hydrothermal vent mussels. *eLife*. **4**, e07966

31. Wentrup, C., Wendeberg, A., Huang, J. Y., Borowski, C. & Dubilier, N. Shift from widespread symbiont infection of host tissues to specific colonization of gills in juvenile deep-sea mussels. *ISME J* **7**, 1244–1247 (2013).

32. Franke, M., Geier, B., Hammel, J. U., Dubilier, N. & Leisch, N. Coming together—symbiont acquisition and early development in deep-sea bathymodioline mussels. *Proc.R. Soc. B*. **288**, 20211044 (2021)

33. Streams, M. E., Fisher, C. R. & Fiala-Médioni, A. Methanotrophic symbiont location and fate of carbon incorporated from methane in a hydrocarbon seep mussel. *Mar. Biol.* **129**, 465–476 (1997).

34. Oishi, S., Moriyama, M., Koga, R. & Fukatsu, T. Morphogenesis and development of midgut symbiotic organ of the stinkbug *Plautia stali* (Hemiptera: Pentatomidae). *Zoological Lett.* **5**, 16 (2019).

**Table S1. Fluorescently labeled probes used in this study.**

| **Probe sequence (5’-3’)** | **Probe** | **Target Group** | **Fluorophore** | **Ref.** |
| --- | --- | --- | --- | --- |
| TTAGCTGCGCCACTAAACCCT | Sym-845 | *Ca.* Thiodiazotropha | Cy-3 DOPE | ^2^ |
| GCTGCCTCCCGTAGGAGT | EUB-I | Most bacteria | Cy-5 DOPE | ^3^ |
| GCAGCCACCCGTAGGTGT | EUB-II | Planctomycetales | Cy-5 DOPE | ^3^ |
| GCTGCCACCCGTAGGTGT | EUB-III | Verrucomicrobiales | Cy-5 DOPE | ^4^ |
| ACTCCTACGGGAGGCAGC | NON 338 | non (negative control) | Cy-3 DOPE | ^2^ |

**Table S2. *In silico* probe specificity results for probe SYM-845 using TestProbe 3.0**

Description of columns: Taxa: Organisms listed at the genus level. Coverage: The percentage of the subject (Taxa) sequence that the reference sequence (FISH probe) matches. Specificity: The percent of base pairs that match between the subject sequence and reference sequence. Acc: (Accession number): the number of possible sequences that match the FISH probe. Eligible: The number of sequences that are valid in the accession numbers. Match: The number of valid sequences that match. Mismatch: The number of sequences that do not match. # 16S rRNA reads (V3-V4): The number of sequences from these taxa that were identified in our 16S rRNA reads.

| **Taxa** | **Coverage** | **Specificity** | **Acc.** | **Eligible** | **Match** | **Mismatch** | **# 16S rRNA reads (V3-V4)** |
| --- | --- | --- | --- | --- | --- | --- | --- |
| *Chromatiaceae;*  *Phaeochromatium* | 100 | 100 | 2 | 2 | 2 | 0 | 0 |
| Gammaproteobacteria;  SZB30 | 90.0 | 100 | 10 | 10 | 9 | 1 | 0 |
| *Chromatiaceae*;  *Marichromatium* | 87.5 | 100 | 16 | 16 | 14 | 2 | 0 |
| *Sedimenticolaceae;*  *Ca*. Thiodiazotropha | 77.8 | 100 | 18 | 18 | 14 | 4 | 80,366 |
| *Chromatiaceae;*  *Halochromatium* | 71.0 | 100 | 69 | 69 | 49 | 20 | 0 |
| Nitrococcales;  Nitrococcaceae | 70.0 | 100 | 10 | 10 | 7 | 3 | 0 |
| *Chromatiaceae;* uncultured | 65.4 | 100 | 26 | 26 | 17 | 9 | 0 |
| Gammaproteobacteria;  TDNP-Wbc97-11-7-16 | 50.0 | 100 | 2 | 2 | 1 | 1 | 0 |
| *Chromatiaceae;*  *Lamprocystis* | 38.5 | 100 | 13 | 13 | 5 | 8 | 0 |
| Gammaproteobacteria;  *Endothiovibrio* | 33.3 | 100 | 3 | 3 | 1 | 2 | 0 |

**Figure S1: Top 10 most abundant ASVs in the different tissues of fresh *L. orbiculatus* individuals in spring and fall through 16S rRNA gene sequencing (V4-V5 region).**

(A) Bubble plot depicting the relative abundance (RA%) per sample of the top 10 most abundant ASVs. Only samples with at least 150 reads are shown, samples below this threshold have been removed. (B) Schematic representation of the match between *Ca*. Thiodiazotropha ASVs from both primer pairs - confirmed *in silico* using BLAST (see Methods). The region targeted by the FISH probe SYM-845 is indicated.

# Figure S2: Location of *Ca.* Thiodiazotropha in starved *L*. *orbiculatus* gill and digestive tract. (A) Brightfield 50 µm micrograph. (B) DAPI (cyan) with labeled tissues: Gill, and digestive tract with white arrowheads. (C) DAPI + Sym-845-Cy3 probe (red) (D) DAPI + EUB-mix-Cy5 (blue) white arrowheads highlight EUB-mix probe in the digestive tract. (E) DAPI + Sym-845 + EUB-mix, white arrowheads highlight the blue EUB-mix signal with no Sym-845 signal visible. (F) Sym-845 probe. (G) EUB-mix probe. (H) Sym-845 probe + EUB-mix probe. (I) Brightfield 50 µm micrograph of consecutive tissue section. (J) DAPI probe. (K) DAPI + non-338-Cy3 probe (red). (L) Non-338-Cy3 probe.

**Figure S3. Gills of  *L*. *orbiculatus*.** (A) DAPI overlapping with SYM845-Cy3 (red) probe. (B) DAPI (cyan), showing host DNA and symbiont DNA. (C) Sym845-Cy3  probe. (D) Brightfield. (E) DAPI + Non-338-Cy3 (red). (F) Non-338-Cy3.

# Figure S4: Location of *Ca.* Thiodiazotropha in fresh *L*. *orbiculatus* gill and digestive tract. (A) Brightfield 50 µm micrograph. (B) DAPI (cyan) with labeled tissues: Gill and digestive tract. (C) DAPI + Sym-845-Cy3 probe (red) (D) DAPI + EUB-mix-Cy5 (blue) (E) DAPI + Sym-845 + EUB-mix, white arrowheads highlight the blue EUB-mix signal with no Sym-845 signal visible. (F) Sym-845 probe. (G) EUB-mix probe. (H) Sym-845 probe + EUB-mix probe. (I) DAPI + Non-338-Cy3 (J) Non-338-Cy3.

All micrographs have a white box with the corresponding lower-case letter highlighting the region of interest in Figure S5.

# Figure S5: Location of *Ca.* Thiodiazotropha in fresh *L*. *orbiculatus* digestive tract. (A) Brightfield 50 µm micrograph. (B) DAPI (cyan) with white arrowheads indicating symbiont DAPI signal. (C) DAPI + Sym-845-Cy3 probe (red) with white arrowheads indicating symbiont Sym-845 signal in the digestive tract (D) DAPI + EUB-mix-Cy5 (blue) white arrowheads highlight EUB-mix probe in the digestive tract. (E) DAPI + Sym-845 + EUB-mix, white arrowheads highlight the blue EUB-mix signal with no Sym-845 signal visible. (F) Sym-845 probe. (G) EUB-mix probe. (H) Sym-845 probe + EUB-mix probe. (I) DAPI + non-338-Cy3 probe (red). (J) Non-338-Cy3 probe (K) Brightfield 50 µm micrograph of consecutive tissue section. (L) DAPI

# Figure S6: Bacterial diversity and community structure in fresh *L. orbiculatus* tissues: (A) Bacterial diversity - as indicated by the Shannon index - across the different tissues. Significant differences between tissues or between seasons in the same tissue were assessed through pairwise Wilcox-tests. P-values from multiple testing were adjusted for the false discovery rate using the Benjamin-Hochberg method. Significant differences (i.e. adjusted p-values < 0.05) are depicted by an asterisk. (B) Ordination depicting the differences in bacterial community structure across different tissues (colors) and between seasons for the same tissue (shapes), as assessed through a PERMANOVA analysis.
